# *Fusobacterium nucleatum* Lipopolysaccharides Disrupt the Interaction of Siglec‑7 to Sialoglycans Expressed on Mammalian Cells

**DOI:** 10.1021/jacsau.5c01502

**Published:** 2026-03-04

**Authors:** Manasik Gumah Adam Ali, Venetia Psomiadou, Marta Tiemblo Martín, Dimitra Lamprinaki, Ferran Nieto-Fabregat, Klaudia Sobczak, Matthew S. Macauley, June Ereño-Orbea, Cristina De Castro, Alba Silipo, Thomas J. Boltje, Nathalie Juge

**Affiliations:** † Quadram Institute Bioscience, Norwich Research Park, Norwich NR4 7UQ, United Kingdom; ‡ Synthetic organic chemistry, Institute for molecules and materials, 6029 Radboud University, Heyendaalseweg 135, Nijmegen 6525 AJ, The Netherlands; § Department of Chemical Sciences, University of Naples Federico II, Naples 80131, Italy; ∥ Chemical Glycobiology Lab, 73038 Center for Cooperative Research in Biosciences (CIC bioGUNE), Basque Research and Technology Alliance (BRTA), Bizkaia Technology Park, Building 800, Derio, Bizkaia 48160, Spain; ⊥ Departments of Chemistry, and Medical Microbiology and Immunology, 3158 University of Alberta, Edmonton, Alberta AB T6G 2E1, Canada

**Keywords:** *Fusobacterium nucleatum*, Siglec-7, lipopolysaccharide, sialic acid, colorectal cancer, bioorthogonal chemistry

## Abstract

Sialic acid-binding immunoglobulin-like lectins (Siglecs) are key immune receptors that bind to cell surface sialic acids, leading to modulation of the immune system. Interrupting the Siglec–sialoglycan binding in cancer has been proposed as a potential antitumor response strategy. We previously showed that colon-cancer-associated *Fusobacterium nucleatum* (Fn) ATCC 51191 interacts with Siglec-7 via its lipopolysaccharide (LPS), revealing Fn LPS as a new ligand for Siglec-7. Here, we used glycoengineered cells carrying sialic acid variants to investigate the capacity of LPS isolated from *F. nucleatum* strains to disrupt the interaction between sialic acid expressed on mammalian cells and Siglec-7. We first showed that LPS extracted from *Fusobacterium polymorphum* ATCC 10953, *F. nucleatum* ssp. *animalis* ATCC 51191, and *F. nucleatum* ssp. *nucleatum* ATCC 25586 strains bound to recombinant Siglec-7 in vitro, while no binding was observed with the Siglec-7R124A mutant, suggesting that the binding occurred through the carbohydrate binding V-set domain. Using glycoengineered Jurkat cells and HEK293T cells carrying modified sialic acid forms, we demonstrated that *F. nucleatum* LPS could significantly disrupt the binding of Siglec-7 to these cells and that this inhibition was decreased following neuraminidase treatment, confirming that the interaction between Fn LPS and Siglec-7 is carbohydrate-mediated. We further validated these data using Jurkat cells and HEK293T cells expressing high-affinity sialic acid ligands. We showed that *F. nucleatum* LPS significantly disrupted the binding of Siglec-7 to these cells in a specific manner. These findings offer novel insights into the development of glycomimetic approaches for limiting colon cancer progression.

## Graphical abstract

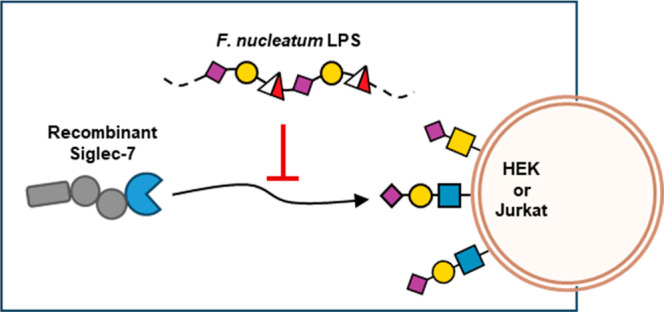

## Introduction

Siglecs (sialic acid-binding immunoglobulin-like lectins) are a family of membrane proteins that function as immune regulatory receptors, recognizing sialylated glycans found on the same cells (cis ligands) or on other cells (trans ligands).^1^ The majority of Siglecs carry immunoreceptor tyrosine-based inhibitory motifs (ITIMs), delivering inhibitory signals upon ligand recognition. These glyco-immune checkpoints have been proposed as new targets for cancer immunotherapy,^2,3^ and interrupting Siglec–sialoglycan binding in cancer could help prevent immune evasion by tumor cells.^4,5^ Siglecs recognize sialic acid-containing glycans through their extracellular domain (ECD) or carbohydrate recognition domain (CRD), which has a homologous N-terminal V-set and a variable number of C2-set Ig-like domains.^6^ Although the CRDs of most Siglecs can recognize sialylated structures, several Siglecs have a broad and overlapping ligand specificity.^7,8^

Among the family of Siglecs, Siglec-7 has been considered to be a target molecule for immunotherapy in cancer due to its potential role in sialic acid-dependent protection of carcinoma killing by NK cells.^9^ Siglec-7 is also expressed by monocytes, where antibody cross-linking can promote inflammatory responses.^10,11^ The enzymatic removal of sialic acids from cancer cell surfaces has been shown to enhance immune cell-mediated clearance of these cells through the loss of Siglec-7 and Siglec-9 binding in cis.^12,13^ In addition, the tumor immune-suppressive effect of Siglec-7 was demonstrated in vivo,^14,15^ further supporting the proposed role of Siglec-7 as an immune checkpoint receptor. However, the range of physiological ligands for Siglec-7 remains to be identified.^8^

Siglec-7 CRD is constituted of two binding sites, a canonical domain characterized by arginine (R124), which interacts with the sialic acid carboxylate, and tryptophan (Trp132), which provides a hydrophobic interaction.^16,17^ Siglec-7 preferentially recognizes α2,8-linked disialic acids, such as those found in gangliosides like GD3, GT1b, and GQ1b, which are highly expressed in neuronal and immune tissues.^18^ To date, GD3,^19^ disialyl Gb5,^20^ disialyl Lewis a, 6-sulfo-Lewis x,^21^ disialyl T,^22^ and Gb3 derivatives^23^ have been reported as sialylated glycan ligands of Siglec-7. Recent studies revealed that Siglec-7 binds to disialyl and trisialyl T structure O-glycans on PDPN, PODXL, and MUC13 on the colon adenocarcinoma cell membrane.^24^ Genome-wide CRISPR screen revealed the glycoprotein CD43 expressed on leukemia cells as a highly specific ligand for Siglec-7, and blocking the interaction relieved Siglec-7-mediated inhibition of immune killing activity.^22^

This inhibitory function can also be targeted by several pathogens that have evolved to express sialic acids on their surfaces to escape host immune responses. For example, Siglec-7 has been shown to interact with *Campylobacter jejuni* in a sialic acid-dependent manner through the rough type LPS, which lacks the *O*-antigen, also known as lipooligosaccharides (LOS) (Avril et al., 2006). Additionally, Siglec-7 interacts in a sialic acid-dependent way with the capsular polysaccharide (CPS) of *Neisseria meningitidis* serogroup Y.^25^ Interestingly, Siglec-7 showed sialic acid-independent binding to β-protein expressed on the Group B Streptococcus surface.^26^

We previously showed that *Fusobacterium nucleatum* (Fn) ATCC 51191, a bacterial species associated with colorectal cancer (CRC), interacts with Siglec-7 via lipopolysaccharide (LPS), revealing Fn LPS as a new ligand for Siglec-7. The binding of *F. nucleatum* ATCC 51191 and its derived LPS induced a pro-inflammatory profile in human monocyte-derived dendritic cells and a tumor-associated profile in human monocyte-derived macrophages.^27^ LPS consists of three distinct domains:^28^ (i) lipid A which is anchored in the bacterial outer membrane, (ii) the core oligosaccharide linked to lipid A, and (iii) the so-called *O*-antigen (or *O*-chain or *O*-specific polysaccharide (OPS), with the latter two pointing to the aqueous environment. The OPS of *F. nucleatum* strains is formed by trisaccharide repeating units and varies in composition among the different strains. The OPS structures of *F. nucleatum* strains show wide strain-specific differences in the trisaccharide repeat unit containing either sialic acid/*N*-acetylneuraminic acid (Neu5Ac),^29^ fusaminic acid,^30^ or monosaccharides other than nonulosonic acid residues.^30–37^ *F. nucleatum* ATCC 51191 OPS contains a linear trisaccharide made up of glucosaminuronic (GlcNAcA and GlcNAc3NAlaA) and fucosamine (FucNAc4N) residues, [→4)-β-D-GlcpNAcA-(1 → 4)-β-D-GlcpNAc3NAlaA-(1 → 3)-α-D-FucpNAc4NR-(1→], with the N-4 of the fucosamine partly acetylated.^34^ STD NMR analyses confirmed the binding of Siglec-7 to *F. nucleatum* ATCC 51191 OPS with contributions from glucosaminuronic (GlcNAcA and GlcNAc3NAlaA) and fucosamine (FucNAc4N) residues.

*F. nucleatum* ssp. *polymorphum* ATCC 10953 and *F. nucleatum* ssp. *nucleatum* ATCC 25586 OPS structures have also been characterized. *F. nucleatum* ssp. *nucleatum* ATCC 25586 OPS consists of a trisaccharide repeating unit, which is phosphorylated with phosphocholine (PCho), and contains Quinovosamine (QuiNAc), 2-acetamido-2,6-dideoxy-l-altrose (L-6dAltNAc), and a novel nonulosonic acid called fusaminic acid (5-acetimidoylamino-3,5,9-trideoxy-gluco-non-2-ulosonic acid) (Non5Am, tentatively assigned to the l-glycero-l-gluco configuration), [→4-β-Non*p*5Am-4-α-L-6dAlt*p*NAc3*P*Cho-3-β-D-Qui*p*NAc-],^33^ and *F. nucleatum* ssp. *polymorphum* ATCC 10953 is made of a trisaccharide repeating unit of α-*N*-acetylneuraminic acid (α-Neu5Ac), β galactose (β galactopyranose), and 2-acetamido-4-amino-2,4,6-trideoxy-a-gal-actopyranose (α-FucNAc4N) residues, [→4)-α-Neu*p*5Ac-(2 → 4)-β-D-Gal*p*-(1 → 3)-α-D-Fuc*p*NAc4N*A*c-(1-], where Ac indicates 4-N-acetylation of ∼30% FucNAc4N residues^29^ (Figure 1A). Recent structural, biophysical, and NMR-guided computational work defined a discrete Siglec-7 epitope within the *O*-antigen of *F. nucleatum* ssp. *polymorphum* ATCC 10953 LPS, centered on an internal Neu5Ac together with 2-acetamido-2,4,6-trideoxy-D-galactose (AAT/FucpNAc4N), providing a molecular basis for Siglec-7 engagement by *F. nucleatum*.^38^

**Figure 1:**
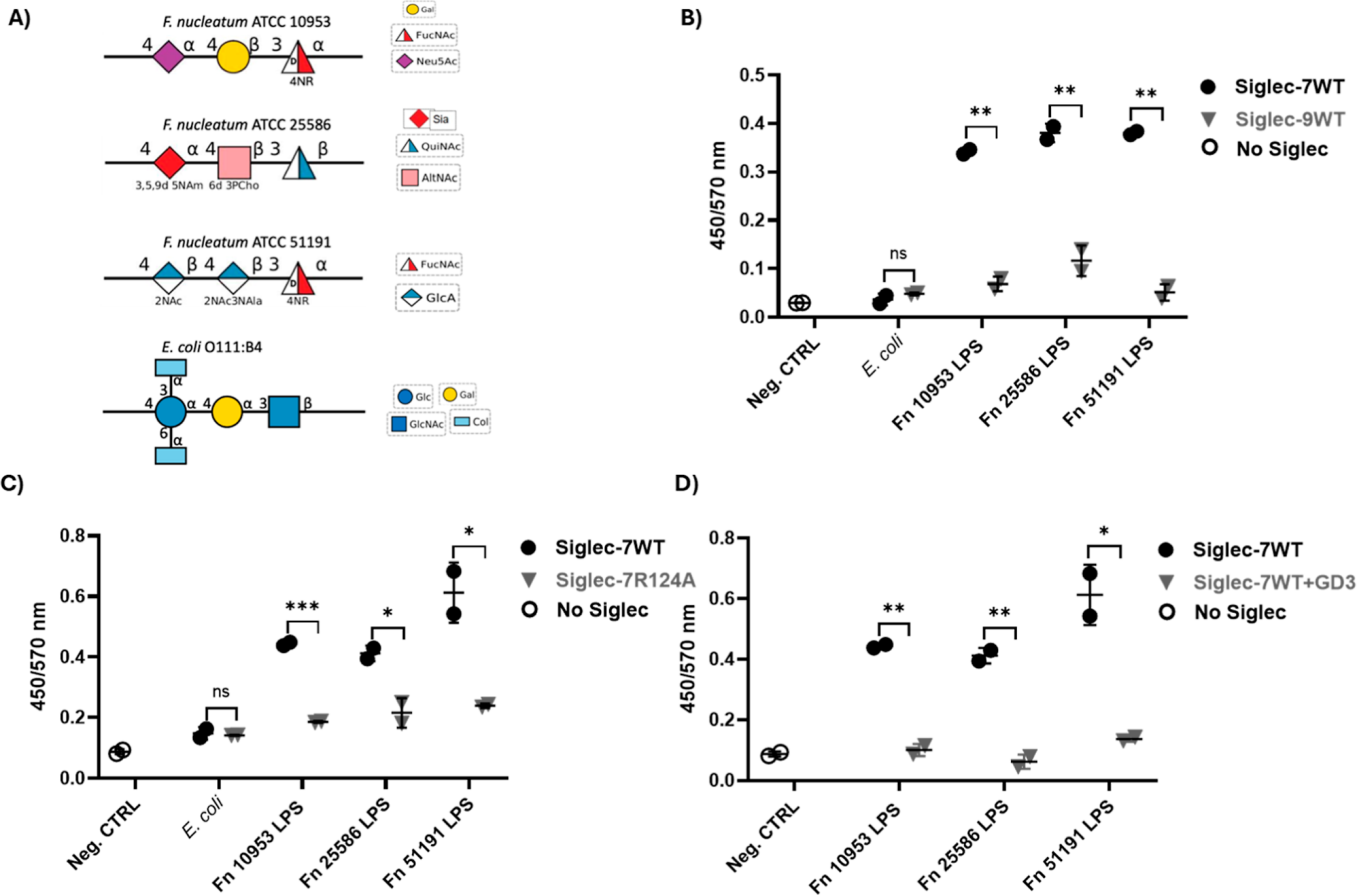
Binding of *F. nucleatum* ssp. purified LPS to Siglecs. (A) Schematic representation of Fn LPS composition from *F. nucleatum* ssp. *polymorphum* ATCC 10953 (Fn 10953),^29^ *F. nucleatum* ssp. *nucleatum* ATCC 25586 (Fn 25586),^33^ *F. nucleatum* ssp. *animalis* ATCC 51191 (Fn 51191),^34^ and *E. coli* O111:B4 ^49^ used as control. (B–D) ELISA-type assays between LPS from Fn 10953, Fn 25586, and Fn 51191 immobilized on the plate and (B) Siglec-7WT and Siglec-9WT, (C) Siglec-7WT and Siglec-7R124A, and (D) Siglec-7WT in the presence or absence of GD3. Siglec-7WT-Fc, Siglec-7R124A-Fc, and Siglec-9WT-Fc were used at 5 μg/mL. *F. nucleatum* LPS was used at 5 μg/mL, *E. coli* LPS at 5 μg/mL, and PBS without Siglec was used as a negative control. GD3 at 5 μg/mL was used for the inhibition assays. Binding was detected by using a spectrophotometer. Statistical analyses were performed using a *t*-test and one-way ANOVA followed by Tukey’s test. *p < 0.05; **p < 0.01; ***p < 0.001; ****p < 0.0001 considered statistically significant; ns, not statistically significant.

Here, we used glycoengineered mammalian cells expressing sialic acid derivatives to investigate the capacity of LPS isolated from *F. nucleatum* ssp. *polymorphum* ATCC 10953, *F. nucleatum* ssp. *animalis* ATCC 51191, and *F. nucleatum* ssp. *nucleatum* ATCC 25586 to disrupt the interaction between sialoglycans expressed on mammalian cells and Siglec-7.

## Materials and Methods

### Materials

*F. nucleatum* ssp. *polymorphum* ATCC 10953, *F. nucleatum* ssp. *animalis* ATCC 51191, and *F. nucleatum* ssp. *nucleatum* ATCC 25586 isolated from clinical isolates were obtained from the American Type Culture Collection (ATCC) in partnership with LGC Standards Ltd. Jurkat cells (TIB-152) and HEK293T cells (CRL-1573) were also from the ATCC. CHO cell line expressing Siglec-7WT-Fc was a kind gift from Paul Crocker (University of Dundee). Recombinant Siglec-7WT-Fc and Siglec-7R124A-Fc proteins purified from CHO cells were from Matthew S Macauley (University of Alberta).^39^ Recombinant Siglec-9WT-Fc and Siglec-9R120A-Fc proteins purified from HEK293 free style (F) cells were from June Ereño-Orbea (CIC bioGUNE).^40^ Sialic acid molecules Ac_5_-SiaNPoc,^41^ Ac_5_-SiaNEtocF,^42^ L4 (benzothiazole derivatives),^43^ and L39 (indole derivatives)^44^ were synthesized as previously reported. *Escherichia coli* O111:B4 LPS used in this study was obtained from Sigma-Aldrich (product code 41202019). All other reagents were purchased from Sigma unless otherwise stated.

### Expression and Purification of Recombinant Siglec-Fc Proteins

CHO-expressing Siglec-7WT-Fc cells were cultured as previously described.^27^ Recombinant Siglec-7WT-Fc was purified by affinity chromatography using KTA FPLC or by Ni2+ affinity chromatography using a 1 or 5 mL HisTrap Excel (GE) column. The protein concentration in each fraction was quantified using a Nanodrop instrument (Thermo Fisher Scientific).

### *F. nucleatum* ssp. Lipopolysaccharide (LPS) Extraction

The extraction of *F. nucleatum*-derived LPS or its derivatives (lipid A, OPS) was performed as previously described.^45^ The OPS domain was further partially depolymerized and purified by gel filtration chromatography, as previously described.^34^

### Enzyme-Linked Immunosorbent Assay (ELISA)-Based Binding Assay

ELISA-based binding assays between Siglecs and *F. nucleatum* LPS were performed as previously described.^27^ Here, Siglec-7WT-Fc (purified from CHO cells), Siglec-7WT-Fc, and Siglec-7R124A-Fc (obtained from Matthew S Macauley) and Siglec-9WT-Fc (obtained from June Ereo-Orbea) were used. Briefly, Fn LPS (10 μg/mL) in 100 mM NaHCO_3_ and 33 mM Na_2_CO_3_ in H_2_O (pH 9) solution were coated on a Nunc MaxiSorp flat-bottom 96-well plate (BioLegend) overnight at 4 °C. Following 3 times washing with 0.05% Tween in PBS, the plate was incubated with 1% bovine serum albumin (BSA) for 1 h at RT. After 3 times of washing, the plates were incubated with precomplexed Siglecs obtained by incubating Siglec-Fc proteins (5 μg/mL) with antihuman IgG Fc-HRP (horseradish peroxidase, Abcam) at 1:50,000 dilution for 1 h at RT. Following incubation for 2 h at room temperature and 3 times washing, the plates were incubated with 3,3′,5,5′-*tetra*-methylbenzidine (TMB) (BioLegend) until color change. Color development was stopped by the addition of 2 N H_2_SO_4_, and the absorbance was measured at 450 nm with a reference of 570 nm using a FLUOStar (BMG Labtech). Data were analyzed using GraphPad Prism 6 Software, Inc., La Jolla, CA. The inhibition assays were carried out as described but in the presence of ganglioside GD3 (Invitrogen) at a concentration of 5 μg/mL. *E. coli* O111:B4 LPS at 10 μg/mL was used as a control.

### Measuring Cell Surface Sialylation Levels

The expression of sialic acids was monitored by flow cytometry following the addition of biotinylated *Maackia amurensis* lectin II (MALII) or *Sambucus nigra* lectin-I (SNA-I) (Invitrogen) (0.04 μg/mL) to the untreated cells or to cells pretreated with sialyltransferase inhibitor Ac_5_-SiaNEtocF at concentrations of 10 μM, and surface sialylation levels were then evaluated using biotinylated lectin (SiaFind Pan-Specific Lectenz Kit, Lectenz Bio) (0.04 μg/mL) followed by streptavidin-PE (2 μg/mL) (Invitrogen, eBioscience). For each condition, cell suspensions were analyzed by flow cytometry, and fluorescence was recorded using the CytoFlex flow cytometer plate-reader function (Beckman Coulter, CA). The setting parameters were collected on a log scale, with more than 10,000 gated events acquired. Experiments were repeated independently on at least three different days using separate cell passages (*n* ≥ 3). Data acquisition and analysis were performed using CytExpert software, version 2.6 (Beckman Coulter). The results were then processed by using Microsoft Excel and GraphPad Prism.

### Chemical Synthesis of Sialic Acid Mimetics and Clickable Ligands

Ac_5_-SiaNPoc,^41^ L4 (benzothiazole derivatives),^43^ L39 (indole derivatives),^44^ and the sialyltransferase inhibitor Ac_5_-SiaNEtocF^42^ were synthesized as previously reported.

### On-Cell Synthesis of Sialic Acid Derivatives

Jurkat cells (TIB-152, ATCC) and HEK293T cells (CRL-1573, ATCC) were cultured in RPMI-1640 or DMEM (Gibco, Thermo Fisher Scientific), respectively, supplemented with 10% heat-inactivated fetal bovine serum (FBS) and 1% penicillin–streptomycin (Gibco, Thermo Fisher Scientific). All cells were maintained at 37 °C in a humidified incubator with 5% CO_2_. To assess the incorporation of clickable sialic acid mimetics (SAMs), cells were incubated for 3 days (72 h) with dimethyl sulfoxide (DMSO) or with increasing concentrations (0.25–512 μM) (Supporting Information Figure 3) or 100 μM (in the rest of the experiments) Ac_5_-SiaNPoc in complete medium. On the day of analysis, HEK293T cells were trypsinized (100 μL of 0.5% Trypsin–EDTA (10×), no phenol red; Thermo Scientific). Both Jurkat and HEK293T cells were transferred to 96-well U-bottom plates (2 × 10^5^ cells/well; Thermo Scientific) and washed three times with 1 × PBS. The cells were then resuspended in 95 μL of freshly prepared click reaction buffer containing 250 μM CuSO_4_ (Sigma-Aldrich), 200 μM THPTA (Tris­(3-hydroxypropyltriazolylmethyl) amine) (Bio-Techne/R&D Systems), and 50 μM azide-PEG_3_-biotin (Sigma-Aldrich) in PBS. To initiate the reaction, 5 μL of 10 mM sodium ascorbate (freshly made, final concentration: 500 μM; Sigma-Aldrich) was added, and the cells were incubated for 30 min at 37 °C. Following the reaction, the cells were washed three times with 100 μL of 1× PBS. To detect incorporation, the cells were stained with 40 μL of the streptavidin–phycoerythrin (PE) conjugate (2 μg/mL; Invitrogen, eBioscience) for 20 min at 4 °C in the dark. Following this, the cells were washed three times with 100 μL of PBA buffer (PBS containing 1% FBS and 0.02% sodium azide), and fluorescence was measured using a CytoFlex flow cytometer (Beckman Coulter, Life Sciences, CA), except for the treatment of Jurkat cells with Ac_5_-SiaNPoc, which was monitored on a Beckman & Dickinson FACS-Calibur flow cytometer. All experiments were independently repeated on different days and with different cell passages to ensure reproducibility (*n* ≥ 3). Data was processed using CytExpert 2.6 (for CytoFlex acquired samples) or FlowJo (FlowJo LLC) (for FACS-Calibur samples) and visualized using GraphPad Prism. The mean fluorescence intensity (MFI) of the PE signal refers to the geometric mean fluorescence intensity value. The establishment of high-affinity Siglec ligand expressing cells (HASLECs) by reacting the alkyne group of the SiaNPoc residues expressed at the cell surface of Jurkat or HEK293T to azides L4 or L39 was carried out as previously described.^46,47^

### Siglec-7 Cell-Based Binding Assays

For Siglec-7-Fc binding assays, glycoengineered cells were centrifuged and washed three times with PBS. The cell pellet was incubated with Siglec-7WT-Fc (purified from CHO cells) at different concentrations (10, 7, 5, 2.5, 1, 0.5 μg/mL) or Siglec-7WT-Fc and Siglec-7R124A-Fc or Siglec-9WT-Fc or Siglec-9R120A-Fc at 1 μg/mL precomplexed with anti-human IgG Fc-FITC antibody (Invitrogen) at 4 μg/mL for 1 h in 100 μL of PBA (1x PBS, 1% FBS and 0.02% sodium azide) at 4 °C. The cells were then washed once with PBA buffer and resuspended in 100 μL of PBA for analysis by flow cytometry. For neuraminidase treatment, the cells were first preincubated for 45 min at 37 °C with 0.25 μM neuraminidase from *Clostridium perfringens* (Invitrogen), washed and resuspended in PBA, and treated with precomplexed Siglec-7WT-Fc-FITC (purified from CHO cells) before analysis.

Siglec-7 binding was assessed using a displacement assay, in which Fn LPS was added after formation of Siglec-7-host ligand complexes. To detect the effect of *F. nucleatum* ssp. purified LPS on Siglec-7 binding to cells, glycoengineered cells were cultured as described above. For the displacement assay, the cells were resuspended in 100 μL with 1 μg/mL precomplex Siglec-7WT-Fc (purified from CHO cells) and anti-human IgG Fc-FITC antibody (Invitrogen) at 4 μg/mL for 1 h in PBA at 4 °C protected from light. The cell pellet was washed once with 100 μL of PBA and incubated with 5 μg/mL *F. nucleatum* LPS or GD3 (as a control) for 40 min at 4 °C in PBS. The cells were then washed three times with PBA and resuspended for flow cytometry analysis. The cell suspensions were analyzed by flow cytometry, and fluorescence was recorded using the CytoFlex flow cytometer plate-reader function (Beckman Coulter, CA). The setting parameters were collected on a log scale with ≥10,000 gated events per condition. All experiments were independently repeated on different days and with different cell passages to ensure reproducibility (*n* ≥ 3). Data were processed with CytExpert 2.6 and visualized using GraphPad Prism. The mean fluorescence intensity (MFI) of the FITC signal refers to the geometric mean fluorescence intensity value.

### Statistical Analyses

Statistical analysis was performed using Prism 5.03 (GraphPad Software, Inc., La Jolla, CA). For comparisons between groups, the *t*-test and one-way ANOVA followed by Tukey’s test were used on GraphPad. *p < 0.05; **p < 0.01; ***p < 0.001; ****p < 0.0001 considered statistically significant; ns, not statistically significant.

## Results

### *F. nucleatum* ssp.-Derived LPS Binds to Recombinant Siglec-7 In Vitro

To investigate the capacity of LPS purified from *F. nucleatum* strains to bind to Siglec-7, LPS of known chemical compositions (Figure 1A) were extracted from *F. nucleatum* ssp. *polymorphum* ATCC 10953, *F. nucleatum* ssp. *animalis* ATCC 51191, and *F. nucleatum* ssp. *nucleatum* ATCC 25586 strains and used in an ELISA-based binding assay (Figure 1B–D). All LPS tested showed significant binding to Siglec-7WT compared to *E. coli* LPS used as a control, while low binding was detected to Siglec-9WT (Figure 1B). Binding to the Siglec-7R124A mutant was significantly reduced (Figure 1C), and a significant reduction in Fn LPS binding was also observed when Siglec-7WT was tested in the presence of ganglioside GD3, a known Siglec-7 ligand^48^ (Figure 1D). Together, these data suggest that Fn LPS binds to Siglec-7WT via the canonical CRD.

### *F. nucleatum* ssp. LPS Significantly Disrupts the Interaction between Siglec-7 and Sialic Acid Mimetics (SAMs) Expressing Cells

Glycoengineered cells were used to test the capacity of Fn LPS to disrupt the interaction between Siglec-7WT and sialic acid expressed by mammalian cells. Sialic acid mimetics (Supporting Information Figure S1) were expressed at the surface of Jurkat cells and HEK293T cells, as previously reported.^46^ Briefly, sialic acids carrying an alkyne on C-5 (Ac_5_-SiaNPoc, 100 μM) were added to human cell cultures and metabolically converted to express cell surface sialoglycans containing an alkyne. Neither Jurkat cells nor HEK293T cells endogenously express Siglecs, minimizing cis interactions during binding assays. Although Jurkat cells lack complex *O*-glycans due to a COSMC mutation (lack of core 1 β3-Gal-T-specific molecular chaperone),^50^ they retain α2,3-sialylated structures that can serve as simplified models for testing trans interactions, as confirmed by MAL-II and SNA-I lectins (Supporting Information Figure S2). SiaNPoc expression in response to Ac_5_-SiaNPoc treatment was monitored by flow cytometry (Supporting Information Figure 3). We selected a Ac_5_-SiaNPoc concentration of 100 μM for further experiments because good metabolic conversion and incorporation and low toxicity was observed at this concentration.

First, Siglec-7WT was titrated using different concentrations (10, 7, 5, 2.5, 1, 0.5 μg/mL), showing dose–response binding to Ac_5_-SiaNPoc-treated Jurkat cells (Supporting Information Figure S4). Siglec-7WT bound to the glycoengineered cells in a sialic acid-dependent manner, as shown by the loss of binding following neuraminidase treatment or using the Ac_5_-SiaNEtocF sialyltransferase inhibitor, confirming that Siglec-7 binding to both Jurkat cells and HEK293T glycoengineered cells is sialic acid-dependent (Figure 2A,B). In addition, binding was significantly reduced with Siglec-7R124A, further supporting Siglec-7 interaction with the cells occurs through the canonical binding site (Figure 2A,B).

**Figure 2:**
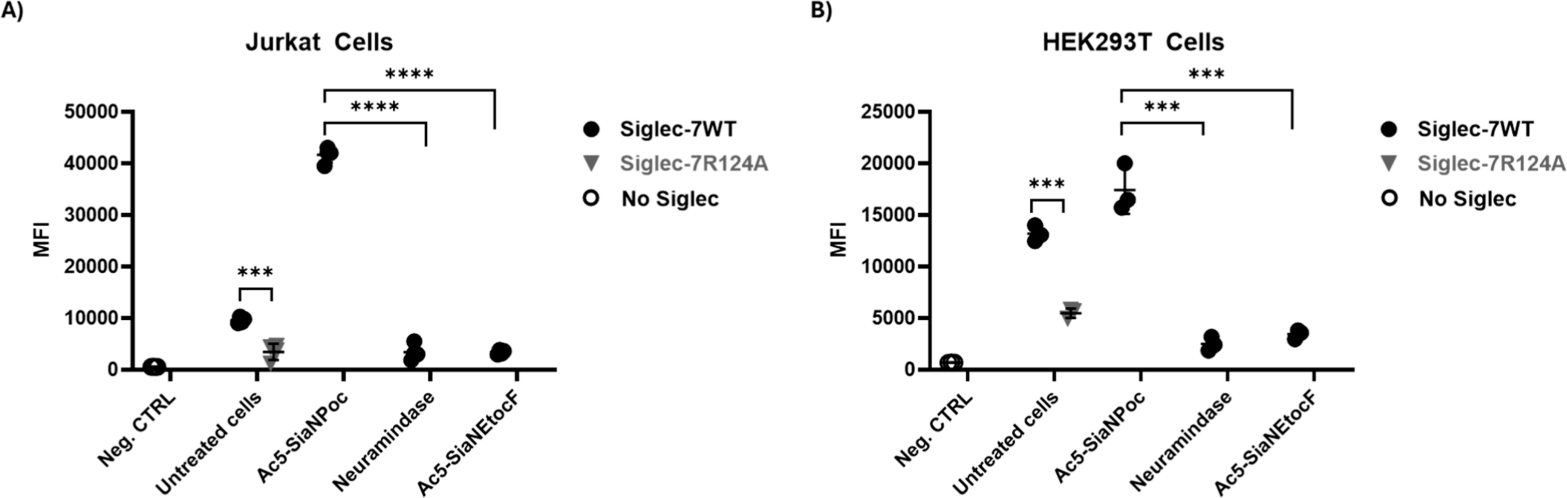
Interaction of Siglec-7 to SAMs. (A) Binding of Siglec-7WT and Siglec-7R124A to untreated, Ac_5_-SiaNPoc (100 μM)-treated, neuraminidase-treated, or Ac_5_-SiaNEtocF (100 μM)-treated Jurkat cells. (B) Binding of Siglec-7WT and Siglec-7R124A to untreated, Ac_5_-SiaNPoc (100 μM)-treated, neuraminidase-treated, or Ac_5_-SiaNEtocF (100 μM)-treated HEK293T cells. Siglec-7WT-Fc and Siglec-7R124A-Fc were used at 1 μg/mL. Untreated cells in PBS (no Siglec) were used as a negative control. Binding was measured by flow cytometry using the CytoFlex flow cytometer plate-reader function (Beckman Coulter, CA) with ≥10,000 gated events per condition. The setting parameters were collected on a log scale. All experiments were independently repeated on different days and with different cell passages to ensure reproducibility (*n* ≥ 3). Data were processed with CytExpert 2.6 software (Beckman Coulter, CA) and visualized using GraphPad Prism. MFI refers to the geometric mean fluorescence intensity value. Statistical analyses were performed using a *t*-test or one-way ANOVA followed by Tukey’s test. *p < 0.05; **p < 0.01; ***p < 0.001; ****p < 0.0001 considered statistically significant; ns, not statistically significant.

We next investigated the effect of *F. nucleatum* LPS as a potential inhibitor of the interaction between Siglec-7WT and Jurkat cells expressing endogenous sialic acid levels using LPS concentrations ranging from 1 to 10 μg/mL (Supporting Information Figure S5) and HEK293T cells expressing endogenous sialic acid levels using LPS concentrations of 1 and 5 μg/mL (Supporting Information Figure S6). The binding was significantly reduced at all concentrations tested, as indicated by quantification analysis of MFI (Supporting Information Figures S5 and S6). We then tested the effect of LPS, OPS or lipid A purified from *F. nucleatum* ATCC 10953, *F. nucleatum* ATCC 25586, and *F. nucleatum* ATCC 51191 on the binding of Siglec-7WT to Ac_5_-SiaNPoc-treated cells (100 μM) (Figure 3A). The results showed significant inhibition of Siglec-7WT binding to Ac_5_-SiaNPoc-treated cells in the presence of *F. nucleatum* LPS and OPS (from the 3 strains) or GD3 used as a control, while no effect was observed with *F. nucleatum* lipid A or *E. coli* LPS. The residual binding is likely due to the design of the inhibition assay, where Siglec-7 was allowed to bind to cell surface ligands prior to the addition of LPS, to better reflect the in vivo situation in the tumor microenvironment. No binding was observed to Siglec-7R124A. Siglec-9WT recognized SAM-expressing cells (albeit at much lower levels than Siglec-7). The binding was significantly reduced in the presence of *F. nucleatum* ATCC 10953, 25586, and 51191 LPS, or with Siglec-9R120A (Figure 3B). Significant inhibition of Siglec-7 binding was also observed in Ac_5_-SiaNPoc-treated HEK293T cells (100 μM) in the presence of *F. nucleatum* LPS and OPS or lipid A with *F. nucleatum* ATCC 10953 and ATCC 51191 (Supporting Information Figure 7A), while no inhibition of Siglec-9 binding was observed using this cell line (Supporting Information Figure 7B).

**Figure 3:**
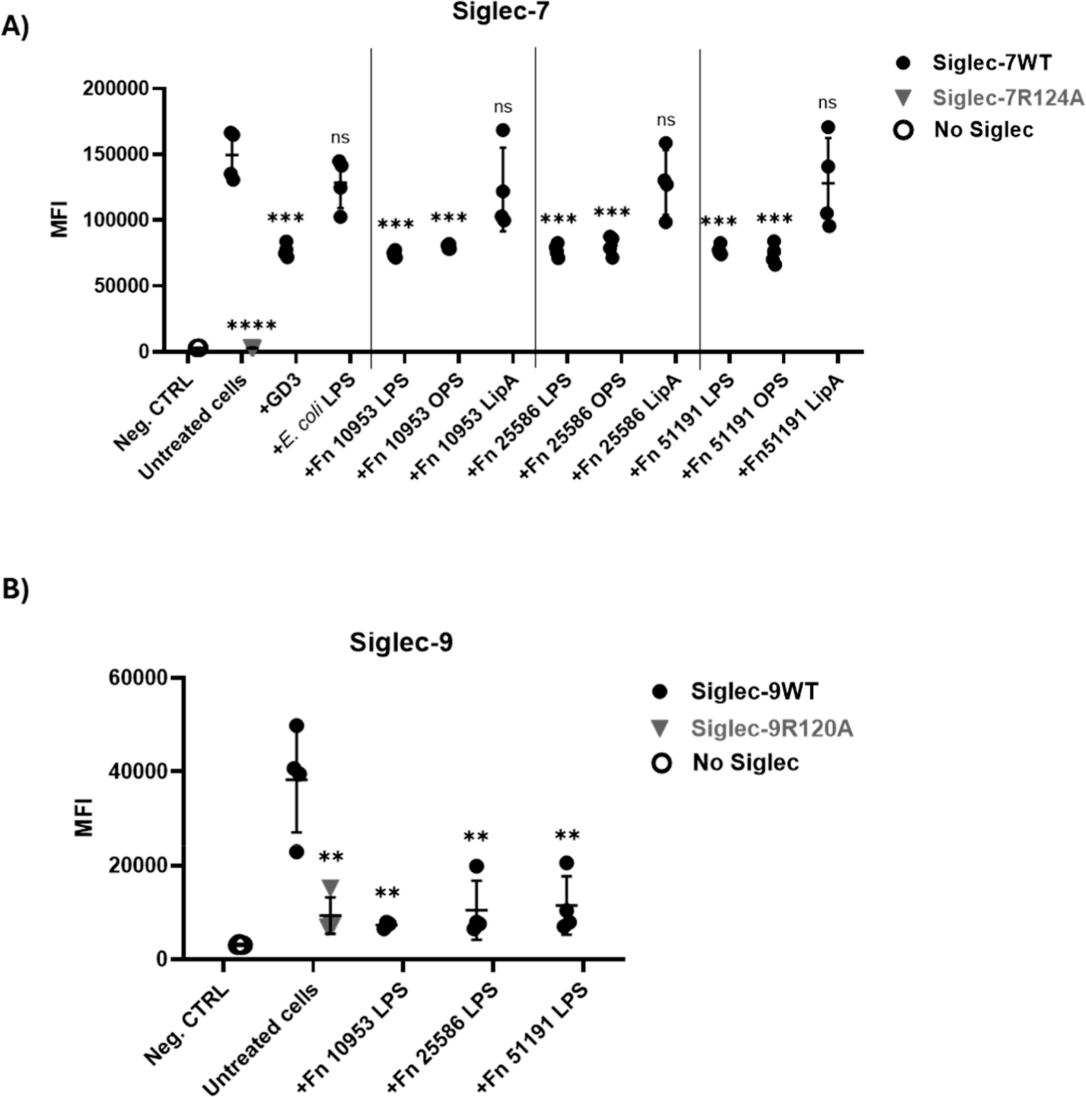
Effect of *F. nucleatum* ssp. LPS on the interaction between Siglecs and SAM-expressing Jurkat cells. Ac_5_-SiaNPoc (100 μM)-treated cells were incubated with Siglec-7R124A or Siglec-7WT in the presence or absence of GD3 (5 μg/mL) and *E. coli* LPS (5 μg/mL) as controls and LPS, OPS, or Lipid A from (A) *F. nucleatum* ATCC 10953, *F. nucleatum* ATCC 25586, or *F. nucleatum* ATCC 51191. Alternatively, cells were incubated with Siglec-9WT or Siglec-9R120A in the presence or absence of (B) *F. nucleatum* ATCC 10953 LPS, *F. nucleatum* ATCC 25586 LPS, or *F. nucleatum* ATCC 51191 LPS. Siglec-7WT-Fc, Siglec-7R124A-Fc, Siglec-9WT-Fc, and Siglec-9R120A-Fc were used at 1 μg/mL. Untreated cells in PBS (no Siglec) were used as a negative control. Binding was measured by flow cytometry using the CytoFlex flow cytometer plate-reader function (Beckman Coulter, CA), with ≥10,000 gated events per condition. The setting parameters were collected on a log scale. All experiments were independently repeated on different days and with different cell passages to ensure reproducibility (*n* ≥ 3). Data were processed with CytExpert 2.6 software (Beckman Coulter, CA) and visualized using GraphPad Prism. MFI refers to the geometric mean fluorescence intensity value. Statistical analyses were performed using a *t*-test. *p < 0.05; **p < 0.01; ***p < 0.001; ****p < 0.0001 considered statistically significant; ns, not statistically significant.

### *F. nucleatum* ssp. LPS Significantly Disrupts the Interaction between the Siglec-7 Interaction and High-Affinity Ligand-Expressing Cells (HASLECs)

We next tested the ability of *F. nucleatum* LPS to disrupt the interaction of Siglec-7 with high-affinity sialic acid ligand expressing cells (HASLECs) (Figure 4). To this end, Jurkat cells were treated with Ac_5_-SiaNPoc which upon metabolic incorporation was reacted with azide compounds using CuAAC click chemistry to generate high-affinity ligands.^46^ These ligands (chemical structures described in Supporting Information Figure S1) have been shown to enhance binding to Siglec-7 on cells.^46^ We first showed that the binding of Siglec-7WT increased upon reacting Ac_5_-SiaNPoc-treated cells with L4 or L39 (Figure 4A). A significant inhibition of binding of Siglec-7WT to Ac_5_-SiaNPoc-treated Jurkat cells was observed with GD3 and *F. nucleatum* ATCC 10953 LPS (Figure 4B). The binding was also reduced in the presence of *F. nucleatum* ATCC 25586 and 51191 LPS, although not statistically significant (*p* = 0.0511 and 0.0528, respectively) (Figure 4B). However, significant inhibition of binding with GD3 and all *F. nucleatum* ssp. LPS was also observed in the high-affinity Siglec ligand expressing cells created upon reaction with L39 (Figure 4C) or L4 (Figure 4D).

**Figure 4:**
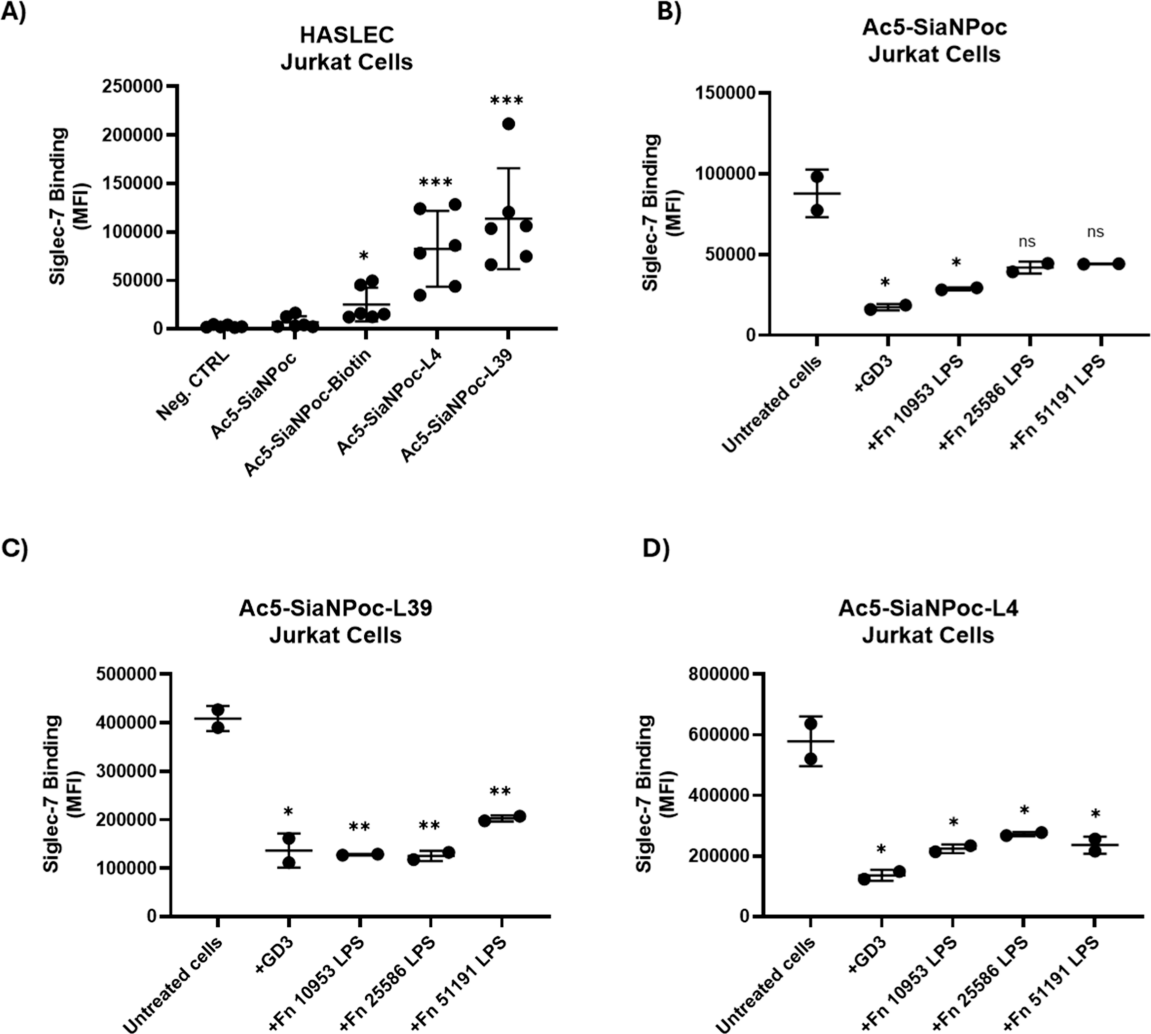
Effect of *F.* *nucleatum* ssp. LPS on the interaction between Siglec-7 and HASLEC Jurkat cells. (A) Interaction between Siglec-7WT and Ac_5_-SiaNPoc-, Ac_5_-SiaNPoc-L4-, and Ac_5_-SiaNPoc-L39-treated Jurkat cells. SiaNPoc-Biotin was used as a control, and cells in PBS without Siglec were used as a negative control. (B–D) Effect of *F. nucleatum* ssp. LPS on the interaction between Siglec-7WT to (B) Ac_5_-SiaNPoc SiaNPoc-treated Jurkat cells, (C) Ac_5_-SiaNPoc-L39-treated Jurkat cells, and (D) Ac_5_-SiaNPoc-L4-treated Jurkat cells. The binding of Siglec-7WT (1 μg/mL) to HASLECs was determined by flow cytometry in the presence or absence of GD3 and LPS from *F. nucleatum* ATCC 10953, ATCC 25586, and ATCC 51191 at 5 μg/mL. Binding was measured by flow cytometry using the CytoFlex flow cytometer plate-reader function (Beckman Coulter, CA) with ≥10,000 gated events per condition. The setting parameters were collected on a log scale. All experiments were independently repeated on different days and with different cell passages to ensure reproducibility (*n* ≥ 3). Data were processed with CytExpert 2.6 software (Beckman Coulter, CA) and visualized using GraphPad Prism. MFI refers to the geometric mean fluorescence intensity value. Statistical analyses were performed using a *t*-test. *p < 0.05; **p < 0.01; ***p < 0.001; ****p < 0.0001 considered statistically significant; ns, not statistically significant.

Siglec-7WT also bound to the high-affinity Siglec ligand expressing HEK293T cells. Ac5-SiaNPoc-treated HEK293T cells were reacted with L4 or L39 to introduce chemically modified sialic acids, which improved Siglec-7WT binding. Binding was significantly reduced with the Siglec-7R124A mutant indicating that binding occurred via the carbohydrate recognition domain (Figure 5A).

**Figure 5:**
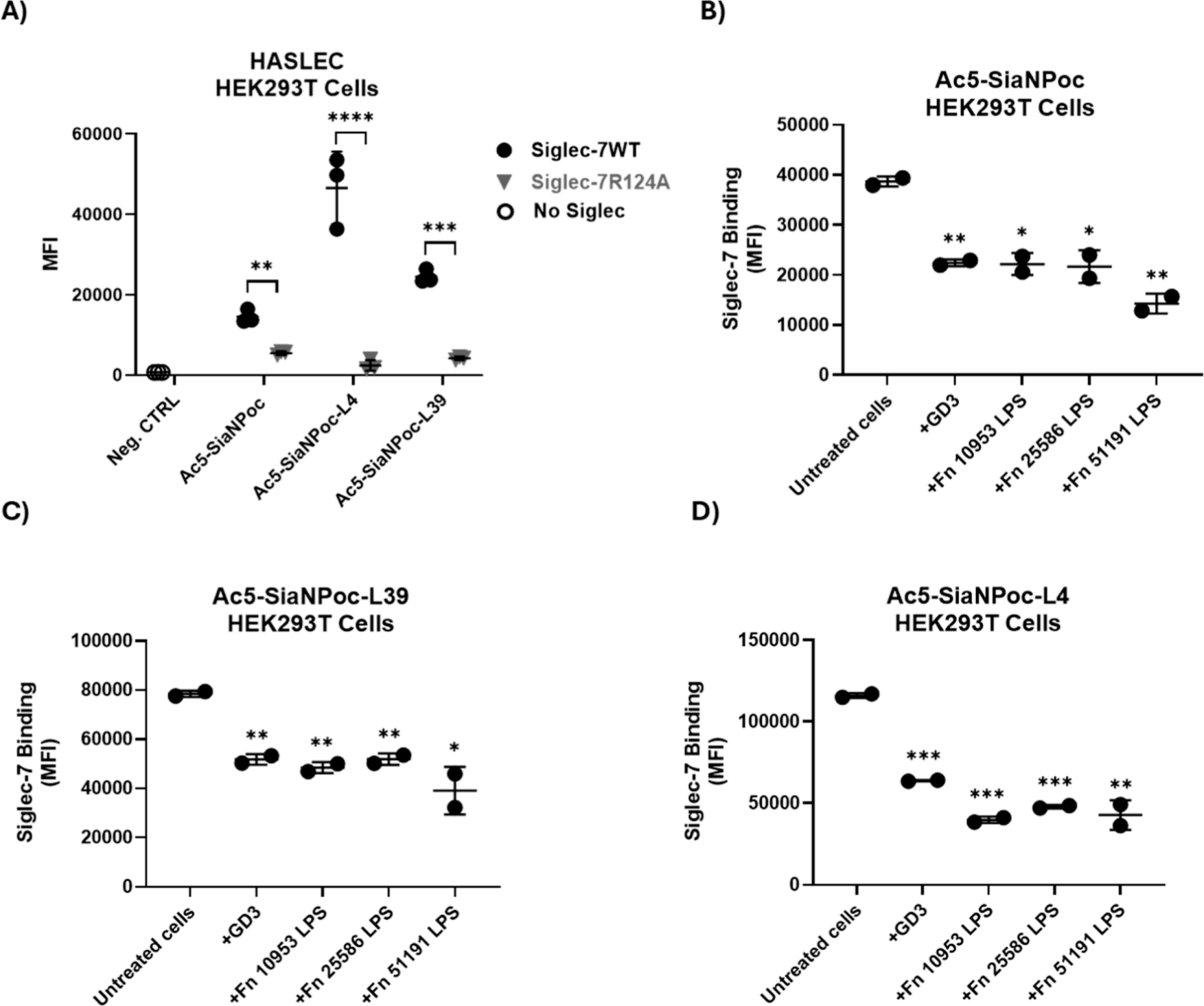
Effect of *F. nucleatum* ssp. LPS on the interaction between Siglec-7 and HASLEC HEK293T cells. (A) Interaction between Siglec-7WT and Ac_5_-SiaNPoc-, Ac_5_-SiaNPoc-L4-, and Ac_5_-SiaNPoc-L39-treated HEK293T cells. Cells in PBS without Siglec were used as a negative control. (B–D) Effect of *F. nucleatum* ssp. LPS or GD3 treatments on the interaction between Siglec-7WT and (B) Ac_5_-SiaNPoc-treated Jurkat cells, (C) Ac_5_-SiaNPoc-L39 treated Jurkat cells, and (D) Ac_5_-SiaNPoc-L4-treated Jurkat cells. The binding of Siglec-7WT (1 μg/mL) or Siglec-7R124A (1 μg/mL) to HASLECs was determined by flow cytometry in the presence or absence of GD3 and LPS from *F. nucleatum* ATCC 10953, ATCC 25586, and ATCC 51191 at 5 μg/mL. Binding was measured by flow cytometry using the CytoFlex flow cytometer plate-reader function (Beckman Coulter, CA), with ≥10,000 gated events per condition. The setting parameters were collected on a log scale. All experiments were independently repeated on different days and with different cell passages to ensure reproducibility (*n* ≥ 3). Data were processed with CytExpert 2.6 software (Beckman Coulter, CA) and visualized using GraphPad Prism. MFI refers to the geometric mean fluorescence intensity value. Statistical analyses were performed using a *t*-test. *p < 0.05; **p < 0.01; ***p < 0.001; ****p < 0.0001 considered statistically significant; ns, not statistically significant.

There was a significant reduction in the presence of GD3 or LPS from *F. nucleatum* ATCC 10953, ATCC 25586, and ATCC 51191 for Ac_5_-SiaNPoc- (Figure 5B), Ac_5_-SiaNPoc-L39- (Figure 5C), and Ac_5_-SiaNPoc-L4-treated cells (Figure 5D).

These data revealed that *F. nucleatum* ssp. LPS disrupts the interaction between Siglec-7 and HASLECs and further confirms the role of *F. nucleatum* LPS in mediating the interaction of *F. nucleatum* strains with Siglec-7.

## Discussion

The Siglec–sialoglycan axis has emerged as an attractive therapeutic target to modulate a broad spectrum of diseases including infection, autoimmunity, and cancer.^3^ We previously revealed that Siglec-7 was a receptor of the interaction between CRC-associated *F. nucleatum* ATCC 51191 and immune cells via LPS,^27^ and this was recently confirmed with *F. nucleatum* ATCC 51191 ^38^ Here, we tested the capacity of LPS extracted from *F. nucleatum* ssp. ATCC 10953, ATCC 25586, and ATCC 51191 strains to disrupt the interaction between Siglec-7 and sialic-acid-expressing mammalian cells. These strains were selected as (i) they are the most clinically relevant subspecies associated with CRC,^51^ influencing immune regulation,^27,38,52^ likely through their engagement with Siglec-7 (as demonstrated for *F. nucleatum* ATCC 10953 and ATCC 51191 strains^27,38^ ), and (ii) their LPS OPS has been structurally characterized.^29,33,34^

We first showed that *F. nucleatum* ATCC 10953-, ATCC 25586-, or ATCC 51191-derived LPS bound to Siglec-7 in vitro and the importance of the Arg124 for the binding of LPS in this interaction. Competitive inhibition assays with GD3 supported the role of the V-set domain in the interaction between Siglec-7 and LPS derived from *F. nucleatum* ATCC 10953, 25586, and ATCC 51191.

The binding affinity between Siglecs and naturally occurring sialylated glycoconjugates is known to be relatively weak (0.1–3 mM).^53^ While electrostatic contributions cannot be entirely ruled out, the lack of binding to *E. coli* LPS, despite its similar anionic charge density, suggests a structural requirement. This is consistent with our NMR findings, which identified unique internal sugar residues in the Fn *O*-antigen that form stable complexes with the Siglec-7 binding pocket.^27,38^ Therefore, biological interactions between Siglecs and their ligands depend on clustering and multivalent binding.^54^

SAMs can feature binding affinities in the nanomolar range.^53,55^ A glycoengineering approach has been developed for on-cell synthesis of SAMs with high affinity for Siglecs on living cells^41,46^ and their multivalent presentation on cell surface glycoproteins and glycolipids. Here, we demonstrated that Jurkat and HEK293T cells treated with sialic acid mimetics, such as Ac_5_-SiaNPoc (SAMs) and high-affinity ligands, such as Ac_5_-SiaNPoc-L4- and Ac_5_-SiaNPoc-L39-treated cells (HASLECs), exhibit significant binding to Siglec-7 compared to unmodified cells. This interaction occurs through the canonical binding site, as shown by using Siglec mutants of canonical binding or through GD3 competition. We confirmed that this interaction occurs in a sialic acid-dependent manner, as shown by the loss of binding following neuraminidase treatment or inhibition of sialylation using Ac_5_-SiaNEtocF.^42^ To investigate glycan–protein interactions, both glycan arrays and cell-based assays offer complementary, but distinct, insights. Glycan arrays enable high-throughput screening of glycan-binding protein specificity across a diverse panel of structurally defined glycans immobilized on a solid surface.^56^ While this format provides quantitative binding data under controlled conditions, it does not capture the complexity of the native glycocalyx. In contrast, cell-based assays present glycans within their natural membrane environment, preserving essential features, such as glycan density, spatial orientation, multivalency, and clustering. These factors can significantly influence binding strength and functional outcomes.^57,58^ Here, we observed that Siglec-7 binding to certain sialic acid mimetics differed markedly from that observed with glycan arrays,^59^ despite using the same ligands and proteins. Enhanced binding was observed in glycoengineered cells (HASLECs), consistent with prior findings demonstrating increased Siglec binding on cells expressing high-affinity ligands, such as Siglec-3, -5, and -14.^46^

We next showed that *F. nucleatum* ssp. ATCC 10953-, ATCC 25586-, and ATCC 51191-derived LPS could significantly disrupt the binding between Siglec-7 and Jurkat cells and HEK293T SAM-expressing cells. Despite the low binding observed between Siglec-9 and Fn LPS in vitro and the lack of Siglec-9 inhibition in the presence of Fn LPS in SAM-expressing HEK293T cells, some level of inhibition was observed in SAM-expressing Jurkat cells. This may reflect differences in glycan density between the two cell lines with HEK293T cells showing higher levels of endogenous sialoglycans compared to Jurkat cells^60^ so that any masking of ligands (due to, e.g., the amphiphilic nature of LPS potentially leading to steric hindrance or aggregation) is exacerbated in the glycan environment presented by SAM-expressing Jurkat cells. In addition, we showed that *F. nucleatum* LPS and OPS but not lipid A could significantly disrupt Siglec-7 interaction with SAM Jurkat cells, suggesting that OPS is the main determinant of the interaction, in line with in vitro STD NMR data for *F. nucleatum* ssp. ATCC 51191 and ATCC 10953.^27,38^ Fn LPS also significantly affected the binding of Siglec-7 to both HASLEC Jurkat and HEK293T cells. The differences in inhibition observed in HASLEC Jurkat and HEK293T cells may arise from a number of factors. First, the sialoglycan type expressed by these two cell lines may differ as mentioned above. Second, the number of SiaNPoc residues that are incorporated and their distribution across the different glycoconjugates may vary among different cell types. Third, the incorporation of SiaNPoc residues may affect the expression levels of glycoconjugates and, therefore, impact Siglec binding. Finally, the bioorthogonal chemistry reaction conditions, reaction efficiency, and the type of reaction partner used for the modification of SiaNPoc residues impact the way Siglecs are able to recognize modified and unmodified sialoglycans. This difference in the inhibition level between HASLEC Jurkat and HEK293T cells was also observed using GD3, supporting the idea that this is inherent to the glycoengineered cell lines rather than a feature of Fn LPS.

Together, our findings demonstrate that *F. nucleatum* LPS OPS is a potent competitive inhibitor of the Siglec-7–sialoglycan axis. Together with the structural characterization of the epitopes engaging in this interaction, these data will inform the development of new targeted glycomimetics inspired by Fn LPS and OPS determinants to modulate the Siglec–sialic acid axis in disease.

## Supplementary Material



## Notes

Figure S1: Chemical structures of the sialic acid mimetics and clickable ligands; Figure S2: Analysis of lectin binding to Jurkat and HEK239T cells; Figure S3: Incorporation of an alkyne sialic acid mimetic into mammalian cells upon treatment with Ac_5_-SiaNPoc; Figure S4: Titration of Siglec-7WT binding to Jurkat cells expressing natural sialic acids or treated with Ac_5_-SiaNPoc; Figure S5: Effect of *F. nucleatum* ssp. LPS on the interaction between Siglec-7WT and Jurkat cells; Figure S6: Effect of *F. nucleatum* ssp. LPS on the interaction between Siglec-7WT and HEK293T cells; and Figure S7: Effect of *F. nucleatum* ssp. LPS on the interaction between Siglecs and HEK293T SAM expressing cells (PDF)

## References

[1] Gianchecchi E., Arena A., Fierabracci A. (2021). Sialic acid-siglec axis in human
immune regulation, involvement in autoimmunity and cancer and potential
therapeutic treatments. Int. J. Mol. Sci..

[2] Feng H., Feng J., Han X., Ying Y., Lou W., Liu L., Zhang L. (2024). The potential of siglecs and sialic
acids as biomarkers
and therapeutic targets in tumor immunotherapy. Cancers.

[3] Läubli H., Nalle S. C., Maslyar D. (2022). Targeting
the siglec–sialic
acid immune axis in cancer: current and future approaches. Cancer Immunol. Res..

[4] Bärenwaldt A., Läubli H. (2019). The sialoglycan-Siglec glyco-immune
checkpoint–a
target for improving innate and adaptive anti-cancer immunity. Expert Opin. Ther. Targets.

[5] van
de Wall S., Santegoets K. C., van Houtum E. J., Büll C., Adema G. J. (2020). Sialoglycans and siglecs can shape
the tumor immune microenvironment. Trends Immunol..

[6] Avril T., Wagner E. R., Willison H. J., Crocker P. R. (2006). Sialic acid-binding
immunoglobulin-like lectin 7 mediates selective recognition of sialylated
glycans expressed on Campylobacter jejuni lipooligosaccharides. Infect. Immun..

[7] Gonzalez-Gil A., Schnaar R. L. (2021). Siglec ligands. Cells.

[8] Gonzalez-Gil A., Li T. A., Kim J., Schnaar R. L. (2023). Human sialoglycan
ligands for immune inhibitory Siglecs. Mol.
Aspects Med..

[9] Hudak J. E., Canham S. M., Bertozzi C. R. (2014). Glycocalyx
engineering reveals a
Siglec-based mechanism for NK cell immunoevasion. Nat. Chem. Biol..

[10] Ikehara Y., Ikehara S. K., Paulson J. C. (2004). Negative regulation of T cell receptor
signaling by Siglec-7 (p70/AIRM) and Siglec-9. J. Biol. Chem..

[11] Varchetta S., Brunetta E., Roberto A., Mikulak J., Hudspeth K. L., Mondelli M. U., Mavilio D. (2012). Engagement of Siglec-7 Receptor Induces
a Pro-inflammatory Response Selectively in Monocytes. PLoS ONE.

[12] Xiao H., Woods E. C., Vukojicic P., Bertozzi C. R. (2016). Precision glycocalyx
editing as a strategy for cancer immunotherapy. Proc. Natl. Acad. Sci. U. S. A..

[13] Daly J., Carlsten M., O’Dwyer M. (2019). Sugar free:
novel immunotherapeutic
approaches targeting siglecs and sialic acids to enhance natural killer
cell cytotoxicity against cancer. Front. Immunol.i.

[14] Ibarlucea-Benitez I., Weitzenfeld P., Smith P., Ravetch J. V. (2021). Siglecs-7/9
function
as inhibitory immune checkpoints in vivo and can be targeted to enhance
therapeutic antitumor immunity. Proc. Natl.
Acad. Sci. U. S. A..

[15] Rodriguez E., Boelaars K., Brown K., Eveline Li R., Kruijssen L., Bruijns S. C., van Ee T., Schetters S. T., Crommentuijn M. H., van der Horst J. C., van Grieken N. C. T., van Vliet S. J., Kazemier G., Giovannetti E., Garcia-Vallejo J. J., van Kooyk Y. (2021). Sialic acids in pancreatic cancer
cells drive tumour-associated macrophage differentiation via the Siglec
receptors Siglec-7 and Siglec-9. Nat. Commun..

[16] Alphey M. S., Attrill H., Crocker P. R., van Aalten D. M. (2003). High resolution
crystal structures of Siglec-7: insights into ligand specificity in
the Siglec family. J. Biol. Chem..

[17] Attrill H., Takazawa H., Witt S., Kelm S., Isecke R., Brossmer R., Ando T., Ishida H., Kiso M., Crocker P. R., van Aalten D. M. (2006). The structure
of siglec-7 in complex
with sialosides: leads for rational structure-based inhibitor design. Biochem. J..

[18] Mohan, B. Role of Siglec-7 in Ganglioside Recognition and Modulating NK Cell Biology; University of Dundee, 2013.

[19] Nicoll G., Avril T., Lock K., Furukawa K., Bovin N., Crocker P. R. (2003). Ganglioside GD3 expression on target
cells can modulate
NK cell cytotoxicity via siglec-7-dependent and-independent mechanisms. Eur. J. Immunol..

[20] Kawasaki Y., Ito A., Withers D. A., Taima T., Kakoi N., Saito S., Arai Y. (2010). Ganglioside DSGb5, preferred ligand for Siglec-7, inhibits NK cell
cytotoxicity against renal cell carcinoma cells. Glycobiology.

[21] Miyazaki K., Sakuma K., Kawamura Y. I., Izawa M., Ohmori K., Mitsuki M., Yamaji T., Hashimoto Y., Suzuki A., Saito Y., Dohi T., Kannagi R. (2012). Colonic epithelial
cells express specific ligands for mucosal macrophage immunosuppressive
receptors siglec-7 and-9. J. Immunol..

[22] Wisnovsky S., Möckl L., Malaker S. A., Pedram K., Hess G. T., Riley N. M., Gray M. A., Smith B. A., Bassik M. C., Moerner W., Bertozzi C. R. (2021). Genome-wide CRISPR screens reveal
a specific ligand for the glycan-binding immune checkpoint receptor
Siglec-7. Proc. Natl. Acad. Sci. U. S. A..

[23] Di
Carluccio C., Padilla-Cortés L., Tiemblo-Martìn M., Gheorghita G. R., Oliva R., Cerofolini L., Masi A. A., Abreu C., Tseng H. K., Molinaro A., Del Vecchio P., Vaněk O., Lin C. C., Marchetti R., Fragai M., Silipo A. (2025). Insights into Siglec-7 Binding to
Gangliosides: NMR Protein Assignment and the Impact of Ligand Flexibility. Advanced Science.

[24] Hashimoto N., Ito S., Harazono A., Tsuchida A., Mouri Y., Yamamoto A., Okajima T., Ohmi Y., Furukawa K., Kudo Y., Kawasaki N., Furukawa K. (2024). Bidirectional signals generated by
Siglec-7 and its crucial ligand tri-sialylated T to escape of cancer
cells from immune surveillance. iScience.

[25] Di
Carluccio C., Gerpe Amor T., Lenza M. P., Masi A. A., Abreu C., Longo V., Albano F., Nieto-Fabregat F., Salvatore P., Falco G., Santana-Medero D., Fragai M., van Kooyk Y., Molinaro A., Valdes-Balbin Y., Vaněk O., Verez-Bencomo V., Marchetti R., Chiodo F., Silipo A. (2025). Molecular Basis of Siglec-7 Recognition
by Neisseria meningitidis Serogroup Y CPS: Implications for Immune
Evasion. JACS Au.

[26] Fong J. J., Tsai C.-M., Saha S., Nizet V., Varki A., Bui J. D. (2018). Siglec-7 engagement by GBS β-protein suppresses
pyroptotic cell death of natural killer cells. Proc. Natl. Acad. Sci. U. S. A..

[27] Lamprinaki D., Garcia-Vello P., Marchetti R., Hellmich C., McCord K. A., Bowles K. M., Macauley M. S., Silipo A., De Castro C., Crocker P. R., Juge N. (2021). Siglec-7 Mediates
Immunomodulation
by Colorectal Cancer-Associated Fusobacterium nucleatum ssp. animalis. Front. Immunol..

[28] Alexander C., Rietschel E. T. (2001). Invited
review: Bacterial lipopolysaccharides and innate
immunity. J. Endotoxin Res..

[29] Vinogradov E., Michael F. S., Homma K., Sharma A., Cox A. D. (2017). Structure
of the LPS O-chain from Fusobacterium nucleatum strain 10953, containing
sialic acid. Carbohydr. Res..

[30] Vinogradov E., St Michael F., Cox A. D. (2018). Structure of the
LPS O-chain from
Fusobacterium nucleatum strain ATCC 23726 containing a novel 5, 7-diamino-3,
5, 7, 9-tetradeoxy-l-gluco-non-2-ulosonic acid presumably having the
d-glycero-l-gluco configuration. Carbohydr.
Res..

[31] Vinogradov E., St Michael F., Cox A. D. (2018). Structure of the LPS O-chain from
Fusobacterium nucleatum strain MJR 7757 B. Carbohydr.
Res..

[32] Vinogradov E., Michael F. S., Cox A. D. (2017). Structure
of the LPS O-chain from
Fusobacterium nucleatum strain 12230. Carbohydr.
Res..

[33] Vinogradov E., Michael F. S., Cox A. D. (2017). The structure
of the LPS O-chain
of Fusobacterium nucleatum strain 25586 containing two novel monosaccharides,
2-acetamido-2, 6-dideoxy-l-altrose and a 5-acetimidoylamino-3, 5,
9-trideoxy-gluco-non-2-ulosonic acid. Carbohydr.
Res..

[34] Garcia-Vello P., Di Lorenzo F., Lamprinaki D., Notaro A., Speciale I., Molinaro A., Juge N., De Castro C. (2021). Structure
of the O-Antigen and the Lipid A from the Lipopolysaccharide of Fusobacterium
nucleatum ATCC 51191. ChemBioChem.

[35] Vinogradov E., Michael F. S., Cairns C., Cox A. D. (2022). Structure of the
lipopolysaccharide O-antigens from Fusobacterium nucleatum strains
SB-106CP and HM-992 and immunological comparison to the O-antigen
of strain 12230. Carbohydr. Res..

[36] Vinogradov E., St Michael F., Cox A. D. (2022). Structure of the
lipopolysaccharide
O-antigens from Fusobacterium nucleatum strains HM-994, HM-995, HM-997. Carbohydr. Res..

[37] Vinogradov E., St Michael F., Cairns C., Cox A. D. (2022). The structure of
the LPS O-chain from five Fusobacterium nucleatum strains CTX47T,
CC2_6JVN3, CC2_3FMU1, CC2_1JVN3, HM-996, containing alditol and phosphate
in the main chain and development of mouse monoclonal antibodies specific
to the O-antigens. Carbohydr. Res..

[38] Di Carluccio, C. ; Nieto-Fabregat, F. ; Cerofolini, L. ; Abreu, C. ; Padilla-Cortés, L. ; Gheorghita, G. R. ; Masi, A. A. ; Buono, L. ; Gumah Adam Ali, M. ; Lamprinaki, D. ; Molinaro, A. ; Juge, N. ; Smaldone, G. ; Vaněk, O. ; Fragai, M. ; Marchetti, R. ; Silipo, A. Fusobacterium nucleatum Lipopolysaccharides O-Antigen Defines a Novel Siglec-7 Binding Epitope; JACS Au, 2025. 10.1021/jacsau.5c00810 PMC12648312 41311924

[39] Rodrigues E., Jung J., Park H., Loo C., Soukhtehzari S., Kitova E. N., Mozaneh F., Daskhan G., Schmidt E. N., Aghanya V., Sarkar S., Streith L., St Laurent C. D., Nguyen L., Julien J. P., West L. J., Williams K. C., Klassen J. S., Macauley M. S. (2020). A versatile soluble
siglec scaffold
for sensitive and quantitative detection of glycan ligands. Nat. Commun..

[40] Atxabal U., Fernández A., Moure M. J., Sobczak K., Nycholat C., Almeida-Marrero V., Oyenarte I., Paulson J. C., Escosura A. d. l., Torres T., Reichardt N. C., Jiménez-Barbero J., Ereño-Orbea J. (2024). Quantifying
Siglec-sialylated ligand interactions:
a versatile 19 FT 2 CPMG filtered competitive NMR displacement assay. Chem. Sci..

[41] Büll C., Heise T., Beurskens D. l. M., Riemersma M., Ashikov A., Rutjes F. P., van Kuppevelt T. H., Lefeber D. J., den Brok M. H., Adema G. J., Boltje T. J. (2015). Sialic
acid glycoengineering using an unnatural sialic acid for the detection
of sialoglycan biosynthesis defects and on-cell synthesis of siglec
ligands. ACS Chem. Biol..

[42] Heise T., Pijnenborg J. F., Büll C., van Hilten N., Kers-Rebel E. D., Balneger N., Elferink H., Adema G. J., Boltje T. J. (2019). Potent
metabolic sialylation inhibitors based on C-5-modified
fluorinated sialic acids. J. Med. Chem..

[43] Sanphanya K., Wattanapitayakul S. K., Phowichit S., Fokin V. V., Vajragupta O. (2013). Novel VEGFR-2
kinase inhibitors identified by the back-to-front approach. Bioorg. Med. Chem. Lett..

[44] Menozzi C., Dalko P. I., Cossy J. (2006). Concise synthesis of
the (±)-N
b-desmethyl-meso-chimonanthine. Chem. Commun..

[45] Pither, M. D. ; Silipo, A. ; Molinaro, A. ; Di Lorenzo, F. Extraction, Purification, and Chemical Degradation of LPS from Gut Microbiota Strains. In Glycolipids: Methods and Protocols; Springer, 2023; pp 153–179. 10.1007/978-1-0716-2910-9_13 36587078

[46] Büll C., Heise T., van Hilten N., Pijnenborg J. F., Bloemendal V. R., Gerrits L., Kers-Rebel E. D., Ritschel T., den Brok M. H., Adema G. J., Boltje T. J. (2017). Steering
Siglec–sialic acid interactions on living cells using bioorthogonal
chemistry. Angew. Chem., Int. Ed..

[47] Hornikx D. L., Visser E. A., Psomiadou V., Büll C., Boltje T. J. (2023). Engineering the sialome of mammalian
cells with sialic
acid mimetics. STAR Protoc..

[48] Hashimoto N., Ito S., Tsuchida A., Bhuiyan R. H., Okajima T., Yamamoto A., Furukawa K., Ohmi Y., Furukawa K. (2019). The ceramide moiety
of disialoganglioside (GD3) is essential for GD3 recognition by the
sialic acid–binding lectin SIGLEC7 on the cell surface. J. Biol. Chem..

[49] Gupta R. K., Egan W., Bryla D. A., Robbins J. B., Szu S. C. (1995). Comparative
immunogenicity of conjugates composed of Escherichia coli O111 O-specific
polysaccharide, prepared by treatment with acetic acid or hydrazine,
bound to tetanus toxoid by two synthetic schemes. Infect. Immun..

[50] Ju T., Cummings R. D. (2002). A unique molecular
chaperone Cosmc required for activity
of the mammalian core 1 β3-galactosyltransferase. Proc. Natl. Acad. Sci. U. S. A..

[51] Zepeda-Rivera M., Minot S. S., Bouzek H., Wu H., Blanco-Míguez A., Manghi P., Jones D. S., LaCourse K. D., Wu Y., McMahon E. F. ., Park S. N., Lim Y. K., Kempchinsky A. G., Willis A. D., Cotton S. L., Yost S. C., Sicinska E., Kook J. K., Dewhirst F. E., Segata N., Bullman S., Johnston C. D. (2024). A distinct Fusobacterium nucleatum clade dominates
the colorectal cancer niche. Nature.

[52] Galaski J., Rishiq A., Liu M., Bsoul R., Bergson A., Lux R., Bachrach G., Mandelboim O. (2024). Fusobacterium nucleatum subsp. nucleatum
RadD binds Siglec-7 and inhibits NK cell-mediated cancer cell killing. iScience.

[53] Crocker P. R., Paulson J. C., Varki A. (2007). Siglecs and
their roles in the immune
system. Nat. Rev. Immunol..

[54] Büll C., Heise T., Adema G. J., Boltje T. J. (2016). Sialic acid mimetics
to target the sialic acid–Siglec axis. Trends Biochem. Sci..

[55] Prescher H., Schweizer A., Kuhfeldt E., Nitschke L., Brossmer R. (2014). Discovery
of multifold modified sialosides as human CD22/Siglec-2 ligands with
nanomolar activity on B-cells. ACS Chem. Biol..

[56] Briard J. G., Jiang H., Moremen K. W., Macauley M. S., Wu P. (2018). Cell-based
glycan arrays for probing glycan–glycan binding protein interactions. Nat. Commun..

[57] Chen Y., Ding L., Ju H. (2018). In situ cellular
glycan analysis. Acc. Chem. Res..

[58] Hooper, J. I. Elucidating Biophysical and Structural Mechanisms of Multivalent lectin-glycan Binding Using Glyconanoparticle Probes; University of Leeds, 2023.

[59] Büll C., Nason R., Sun L., Van Coillie J., Madriz Sørensen D., Moons S. J., Yang Z., Arbitman S., Fernandes S. M., Furukawa S., McBride R., Nycholat C. M., Adema G. J., Paulson J. C., Schnaar R. L., Boltje T. J., Clausen H., Narimatsu Y. (2021). Probing the binding specificities
of human Siglecs by cell-based glycan arrays. Proc. Natl. Acad. Sci. U. S. A..

[60] Reinke S. O., Bayer M., Berger M., Hinderlich S., Blanchard V. (2011). Analysis of cell surface N-glycosylation of the human
embryonic kidney 293t cell line. J. Carbohydr.
Chem..

