# Supplementary material for: Fusobacterium nucleatum Lipopolysaccharides Disrupt the Interaction of Siglec‑7 to Sialoglycans Expressed on Mammalian Cells
Source: JACS Au. 2026 Mar 4;6(3):1643–53. doi: 10.1021/jacsau.5c01502 (PMC13014188; doi:10.1021/jacsau.5c01502)
Supplement: Supplementary file 1 [file au5c01502_si_001.pdf]

Supporting Information

Fusobacterium nucleatum lipopolysaccharides disrupt the interaction of Siglec-7 to sialoglycans expressed on mammalian cells

Manasik Gumah Adam Ali[1], Venetia Psomiadou[2], Marta Tiemblo Martín[3], Dimitra Lamprinaki[1], Ferran Nieto-Fabregat[3], Klaudia Sobczak[4], Matthew S. Macauley[5], June Ereño-Orbea[4], Cristina De Castro[3], Alba Silipo[3], Thomas J. Boltje[2*], Nathalie Juge[1*]

[1]Quadram Institute Bioscience, Norwich Research Park, Norwich, NR4 7UQ, United Kingdom

[2]Synthetic organic chemistry, Institute for molecules and materials, Radboud University, Heyendaalseweg 135, Nijmegen, 6525 AJ, The Netherlands

[3]Department of Chemical Sciences, University of Naples Federico II, Naples, 80131, Italy

[4]Chemical Glycobiology lab, Center for Cooperative Research in Biosciences (CIC bioGUNE), Basque Research and Technology Alliance (BRTA), Bizkaia Technology Park, Building 800, Derio, Bizkaia, 48160, Spain

[5]Departments of Chemistry, and Medical Microbiology and Immunology, University of Alberta, Edmonton, Alberta, AB T6G 2E1, Canada

* corresponding authors: nathalie.juge@quadram.ac.uk; thomas.boltje@ru.nl

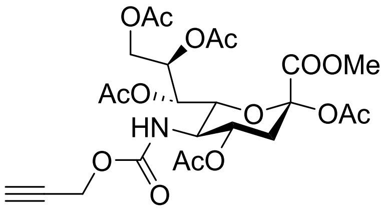

Ac_5-SiaNPoc

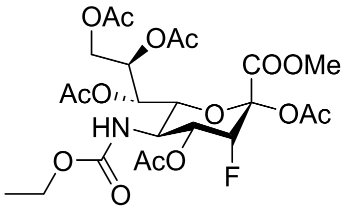

Ac_5-SiaNEtocF

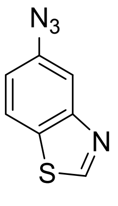

L4

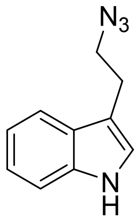

L39

Figure S1. Chemical structures of the sialic acid mimetics and clickable ligands. Ac_5-SiaNPoc[1], Ac_5-SiaNEtocF[2], L4[3] and L39[4] were prepared as previously reported.

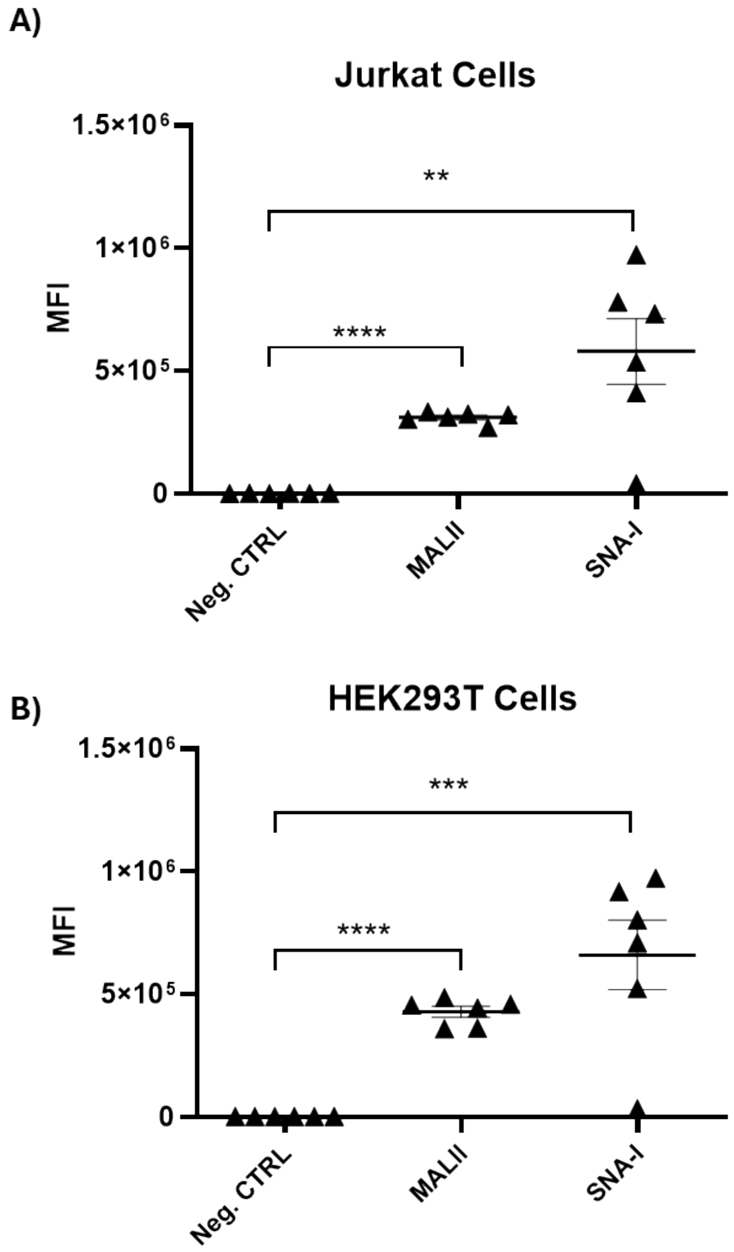

Figure S2. Analysis of lectin binding to Jurkat and HEK239T cells. A) Binding of Jurkat cells to MALII and SNA-I biotinylated lectins. **B)** Binding of HEK293T cells to MALII and SNA-I biotinylated lectins. Untreated cells in PBS were used as a negative control. Cells were incubated with biotinylated lectins (0.04 µg/ml) at 4 °C, followed by staining with streptavidin-FITC (2 µg/ml). Fluorescence was measured by flow cytometry using the CytoFlex flow cytometer equipped with plate-reader function (Beckman Coulter, CA). Fluorescence parameters were collected on a logarithmic scale, and the data were processed using CytExpert 2.6 and visualised with GraphPad Prism. MFI is referred to the geometric mean fluorescence intensity value. Each point represents an individual measurement; horizontal bars indicate the mean MFI. Statistical analyses were performed using a t-test. $p<0.05$; **$p<0.01$; ***$p<0.001$; ****$p<0.0001$ considered statistically significant, ns; not statistically significant.

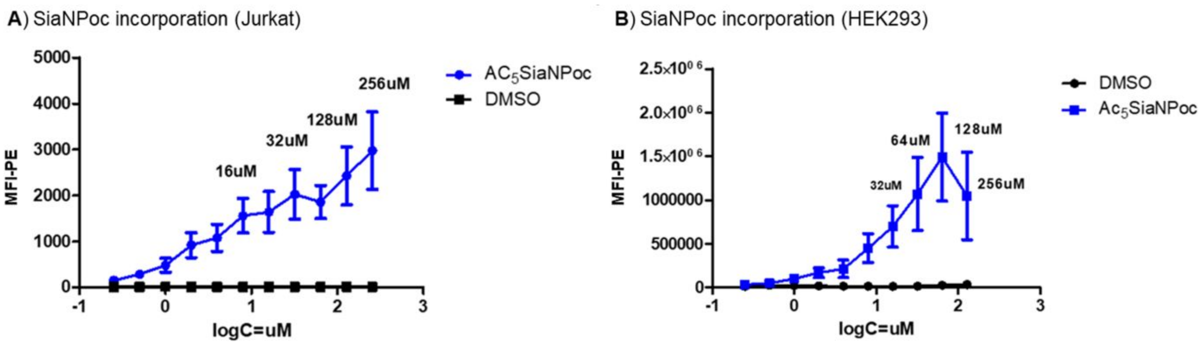

Figure S3. Incorporation of an alkyne sialic acid mimetic into mammalian cells upon treatment with Ac5-SiaNPoc. A) Incorporation of Ac_5SiaNPoc into Jurkat cells at concentrations ranging from 0 to 256 µM. **B)** Incorporation of Ac_5-SiaNPoc into HEK293T cells at concentrations ranging from 0 to 256 µM. For incorporation detection, Ac_5-SiaNPoc modified cells were reacted with biotin-azide followed by incubation with streptavidin-PE (4 µl/ml). Fluorescence was measured by flow cytometry using a Beckman & Dickinson FACS-Calibur flow cytometer for **A** or using the CytoFlex flow cytometer plate-reader function (Beckman Coulter, CA) for **B**. Fluorescence parameters were collected on a logarithmic scale, and the data were processed using CytExpert 2.6 and visualised with GraphPad Prism. MFI is referred to the geometric mean fluorescence intensity value. Each point represents an individual measurement; horizontal bars indicate the mean MFI.

A)

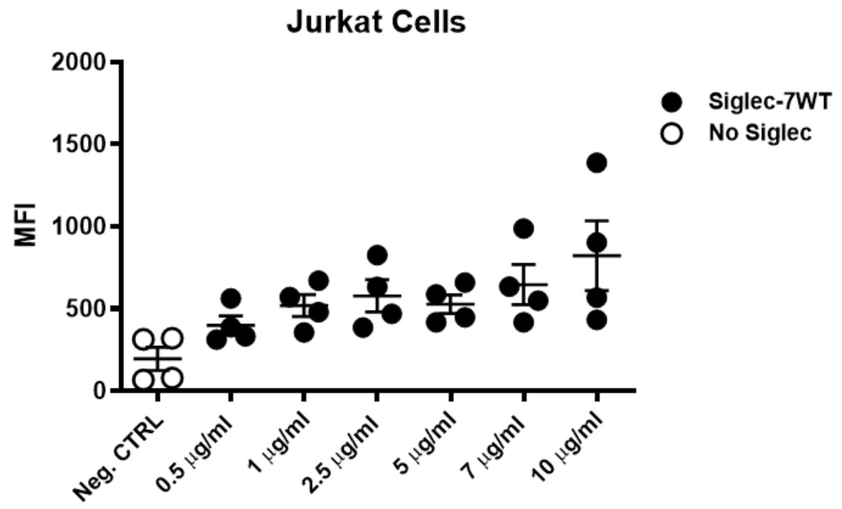

B)

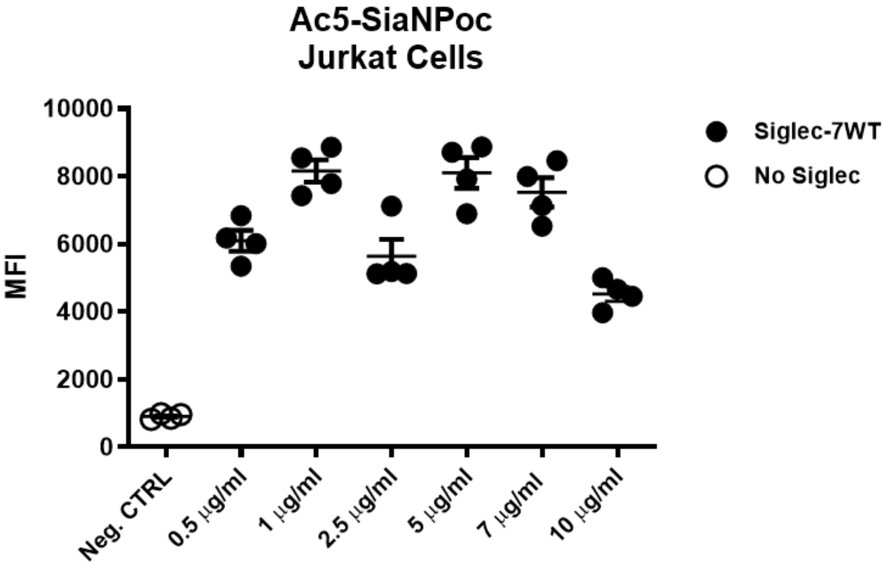

.

Figure S4. Titration of Siglec-7WT binding to Jurkat cells expressing natural sialic acids or treated with Ac5-SiaNPoc. Jurkat cells were cultured in the presence or absence of 100 µM Ac5-SiaNPoc for 3 days at 37°C to allow for the metabolic incorporation of the modified sialic acid mimetic into the cell surface glycome. **A)** Binding of Siglec-7WT to Jurkat cells. **B)** Binding of Siglec-7WT to Ac5-SiaNPoc-treated Jurkat cells. Cells were incubated for 1 h at 4 °C with increasing concentrations of recombinant Siglec-7WT (10, 7, 5, 2.5, 1, and 0.5 µg/ml), pre-complexed for 15 min at 4 °C with anti-human Fc-FITC antibody (final concentration 2–4 µg/ml). Untreated cells in PBS (no Siglec) were used as negative controls. Fluorescence was measured using a CytoFlex flow cytometer with plate-reader function (Beckman Coulter, CA). Parameters were collected on a logarithmic scale, and data were analysed using CytExpert software version 2.6 (Beckman Coulter). Binding was quantified as geometric mean fluorescence intensity (MFI). Each point represents an individual measurement; horizontal bars indicate the mean MFI.

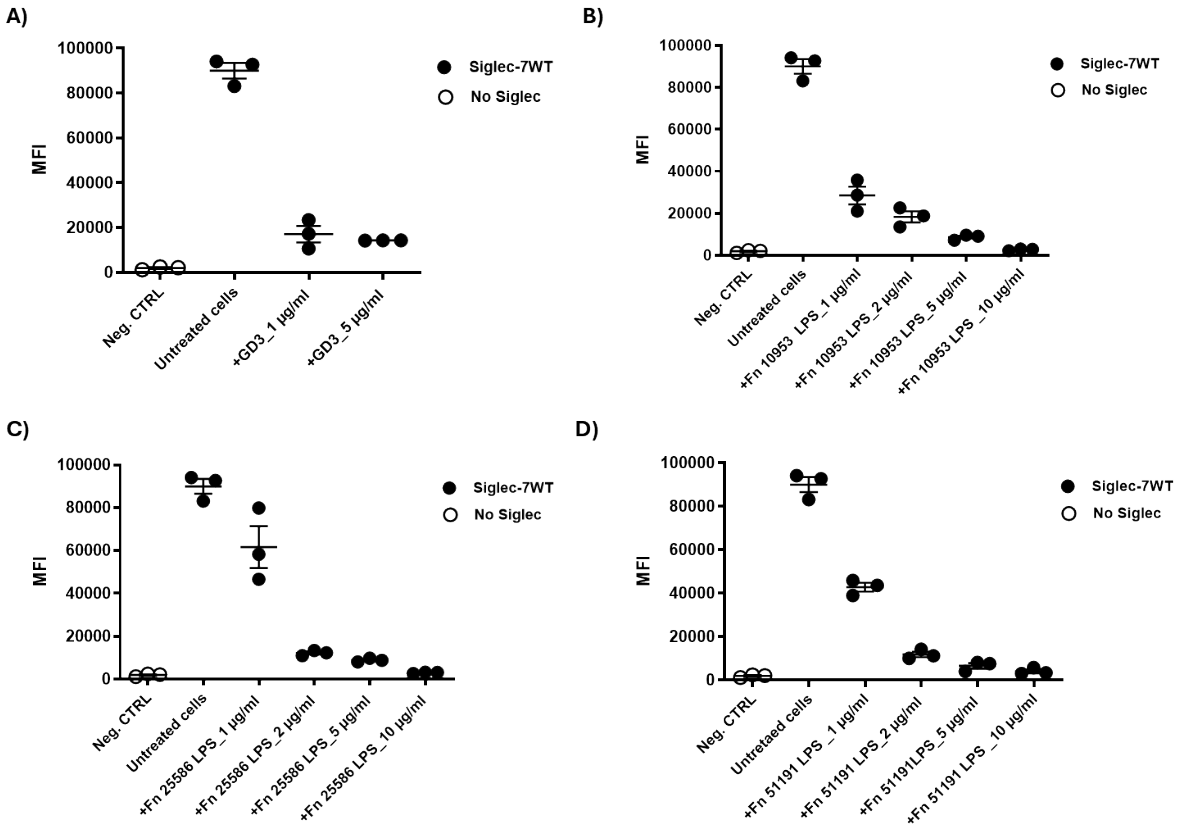

Figure S5. Effect of *F. nucleatum* ssp. LPS on the interaction between Siglec-7WT and Jurkat cells. Jurkat cells expressing endogenous sialic acid levels were incubated with Siglec-7WT in the presence or absence of **A)** GD3 ganglioside (1 or 5 µg/ml, used as a positive control), **B)** *F. nucleatum* ATCC 10953 LPS, **C)** *F. nucleatum* ATCC 25586 LPS, or **D)** *F. nucleatum* ATCC 51191 LPS, each tested at 1, 2, 5, and 10 µg/ml *F. nucleatum* LPS. Siglec-7WT at 1 µg/ml was first pre-complexed with anti-human Fc-FITC antibody at 4 µg/ml for 1 h at 4 °C. Untreated cells in PBS (no Siglec) were used as a negative control. Fluorescence was measured using a CytoFlex flow cytometer with plate-reader function (Beckman Coulter, CA), with settings collected on a logarithmic scale. Data were analysed using CytExpert software version 2.6 (Beckman Coulter). Siglec binding was quantified as geometric mean fluorescence intensity (MFI). Each point represents an individual measurement; horizontal bars indicate the mean MFI.

A)

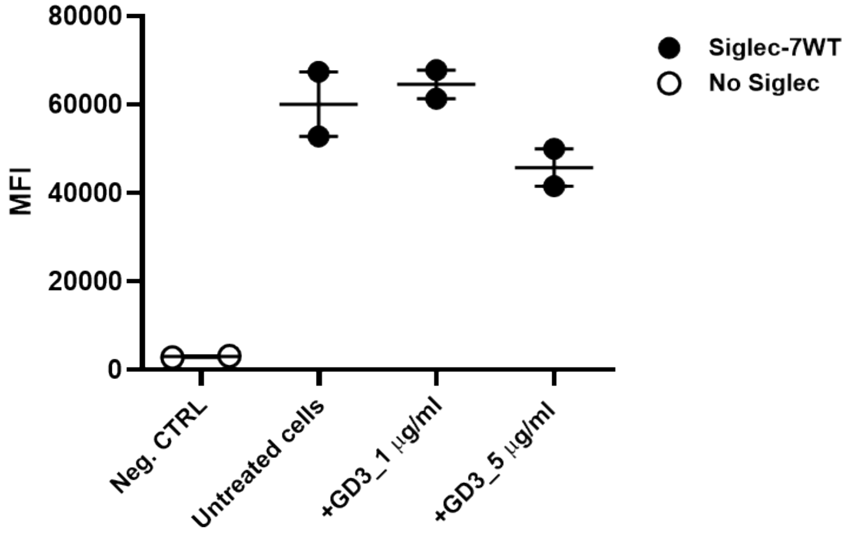

B)

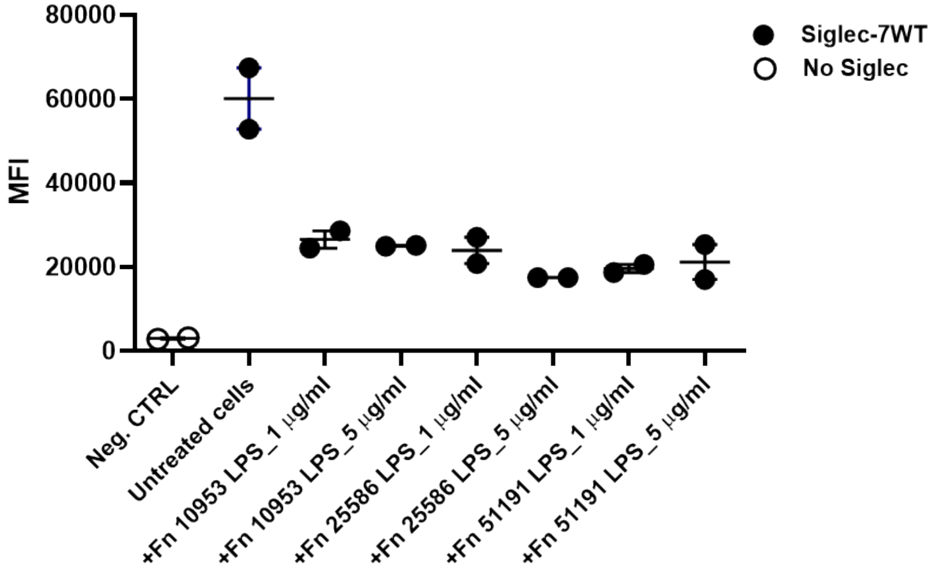

Figure S6. Effect of *F. nucleatum* ssp. LPS on the interaction between Siglec-7WT and HEK293T cells. HEK293T cells expressing endogenous sialic acid levels were incubated with Siglec-7WT in the presence or absence of **A)** GD3 ganglioside (1 or 5 µg/ml, positive control), **B)** *F. nucleatum* ATCC 10953, ATCC 25586, or ATCC 51191 LPS (1 or 5 µg/ml). Siglec-7WT at 1 µg/ml was first pre-complexed with anti-human Fc-FITC antibody at 4 µg/ml for 1 h at 4 °C. Untreated cells in PBS (no Siglec) were used as a negative control. Fluorescence was measured using a CytoFlex flow cytometer with plate-reader function (Beckman Coulter, CA). Parameters were collected on a logarithmic scale, and data were analysed using CytExpert software version 2.6 (Beckman Coulter). Binding was quantified as geometric mean fluorescence intensity (MFI). Each point represents an individual measurement; horizontal bars indicate the mean MFI.

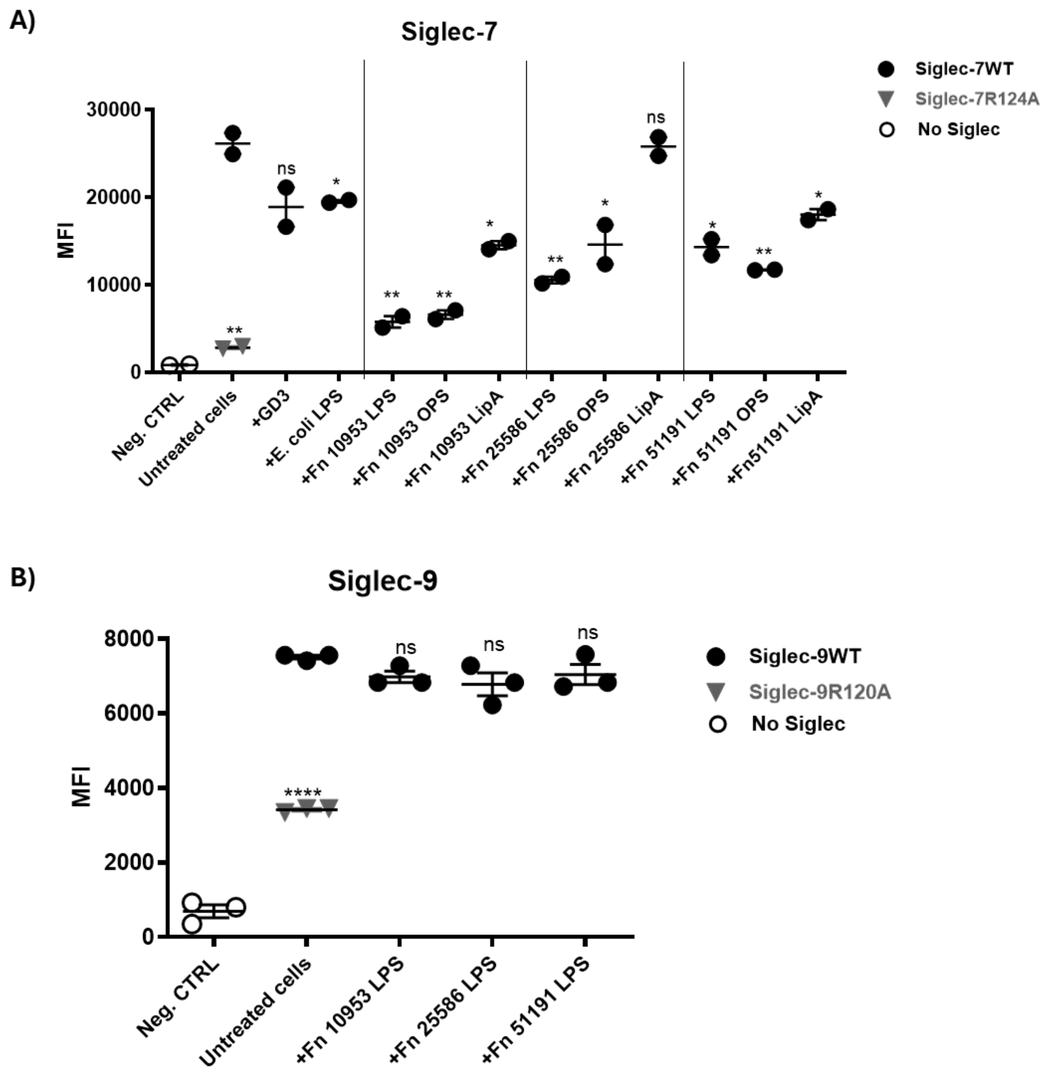

Figure S7. Effect of *F. nucleatum* ssp. LPS on the interaction between Siglecs and HEK293T SAM expressing cells. HEK293T cells were incubated with Siglec7R124A or Siglec-7WT in the presence or absence GD3 (5 µg/ml), *E. coli* LPS (5 µg/ml) as controls and LPS, OPS, or Lipid A from **A)** *F. nucleatum* ATCC 10953 **B)** *F. nucleatum* ATCC 25586 **C)** *F. nucleatum* ATCC 51191. Alternatively, cells were incubated with Siglec-9WT or Siglec-9R120A in the presence or absence of **D)** *F. nucleatum* ATCC 10953 LPS, **E)** *F. nucleatum* ATCC 25586 LPS, **F)** *F. nucleatum* ATCC 51191 LPS. Siglec-7WT or Siglec-7R124A at 1 µg/ml and Siglec-9WT or Siglec-9120A at 1 µg/ml were first pre-complexed with anti-human Fc-FITC antibody at 4 µg/ml. Untreated cells in PBS (no Siglec) were used as a negative control. Fluorescence was measured by flow cytometry using the CytoFlex flow cytometer plate-reader function (Beckman Coulter, CA). The setting parameters were collected on a log scale. The collected data were processed using the cyto-expert software (Beckman Coulter, CA). The MFI of the PE signal is referred to as the geometric mean fluorescence intensity value. Each point represents an individual measurement; horizontal bars indicate the mean MFI. Statistical analyses were performed using a t-test. p<0.05; **p<0.01; ***p<0.001; ****p<0.0001 considered statistically significant, ns; not statistically significant.

References:

(1) Büll, C.; Heise, T.; Beurskens, D. I. M.; Riemersma, M.; Ashikov, A.; Rutjes, F. P.; van Kuppevelt, T. H.; Lefeber, D. J.; den Brok, M. H.; Adema, G. J.; Boltje, T. J. Sialic acid glycoengineering using an unnatural sialic acid for the detection of sialoglycan biosynthesis defects and on-cell synthesis of siglec ligands. *ACS chemical biology* **2015**, *10* (10), 2353-2363.
(2) Heise, T.; Pijnenborg, J. F.; Büll, C.; van Hilten, N.; Kers-Rebel, E. D.; Balneger, N.; Elferink, H.; Adema, G. J.; Boltje, T. J. Potent metabolic sialylation inhibitors based on C-5-modified fluorinated sialic acids. *Journal of medicinal chemistry* **2019**, *62* (2), 1014-1021.
(3) Sanphanya, K.; Wattanapitayakul, S. K.; Phowichit, S.; Fokin, V. V.; Vajragupta, O. Novel VEGFR-2 kinase inhibitors identified by the back-to-front approach. *Bioorganic & medicinal chemistry letters* **2013**, *23* (10), 2962-2967.
(4) Menozzi, C.; Dalko, P. I.; Cossy, J. Concise synthesis of the (±)-N b-desmethyl-meso-chimonanthine. *Chemical Communications* **2006**, (44), 4638-4640.
